# Investigating the role of filamin C in Belgian patients with frontotemporal dementia linked to GRN deficiency in FTLD-TDP brains

**DOI:** 10.1186/s40478-015-0246-7

**Published:** 2015-11-10

**Authors:** Jonathan Janssens, Stéphanie Philtjens, Gernot Kleinberger, Sara Van Mossevelde, Julie van der Zee, Rita Cacace, Sebastiaan Engelborghs, Anne Sieben, Julia Banzhaf-Strathmann, Lubina Dillen, Céline Merlin, Ivy Cuijt, Caroline Robberecht, Bettina Schmid, Patrick Santens, Adrian Ivanoiu, Mathieu Vandenbulcke, Rik Vandenberghe, Patrick Cras, Peter P. De Deyn, Jean-Jacques Martin, Stuart Maudsley, Christian Haass, Marc Cruts, Christine Van Broeckhoven, on behalf of the Belgian Neurology (BELNEU) consortium

**Affiliations:** Neurodegenerative Brain Diseases group, VIB Department of Molecular Genetics, University of Antwerp – CDE, Universiteitsplein 1, B-2610 Antwerp, Belgium; Institute Born-Bunge, University of Antwerp, Antwerp, Belgium; Translational Neurobiology Group, Department of Molecular Genetics, VIB, Antwerp, Belgium; Department of Neurology and Memory Clinic, Hospital Network Antwerp, Middelheim and Hoge Beuken, Antwerp, Belgium; Department of Neurology, University Hospital Ghent and University of Ghent, Ghent, Belgium; German Center for Neurodegenerative Diseases (DZNE), Munich, Germany; Biomedical Center (BMC), Biochemistry, Ludwig-Maximilians University Munich, Munich, Germany; Munich Cluster for Systems Neurology (SyNergy), Munich, Germany; Department of Neurology, Saint-Luc University Hospital and Institute of Neuroscience, Université Catholique de Louvain, Brussels, Belgium; Department of Neurosciences, Faculty of Medicine, KU Leuven, Leuven, Belgium; Department of Old Age Psychiatry and Memory Clinic, University of Leuven, Leuven, Belgium; Department of Neurology, University Hospitals Leuven, Leuven, Belgium; Department of Neurology, Antwerp University Hospital, Edegem, Belgium; Department of Neurology and Alzheimer Research Center, University of Groningen and University Medical Center Groningen, Groningen, The Netherlands

**Keywords:** Filamin C, Genetics, Frontotemporal lobar degeneration, Granulin GRN, Haploinsufficiency, Proteomics

## Abstract

TAR DNA-binding protein 43 (TDP-43) inclusions are pathological hallmarks of patients with frontotemporal lobar degeneration (FTLD) and amyotrophic lateral sclerosis (ALS). Loss of TDP-43 in zebrafish engenders a severe muscle and vascular phenotype with a concomitant elevation of filamin C (FLNC) levels, an observation confirmed in the frontal cortex of FTLD-TDP patients. Here, we aimed to further assess the contribution of FLNC to frontotemporal dementia (FTD) etiology. We conducted a mutational screening of FLNC in a cohort of 529 unrelated Belgian FTD and FTD-ALS patients, and a control cohort of 920 unrelated and age-matched individuals. Additionally we performed an in-depth characterization of FLNC expression levels in FTD patients and a murine FTD model.

In total 68 missense variants were identified of which 19 (MAF < 1 %) were patient-only. Gene burden analysis demonstrated a significant association between the presence of rare variants in FLNC and disease (*P* = 0.0349, RR = 1.46 [95 % CI 1.03–2.07]). Furthermore, elevated FLNC expression levels, observed previously in FTLD-TDP patients, were mainly attributable to FTD patients with the progranulin (GRN) p.0(IVS1 + 5G > C) loss-of-function mutation. Increased FLNC levels were, to a lesser extent, also identified in a *FLNC* p.V831I variant carrier and in FTD patients with the p.R159H mutation in valosin-containing protein (VCP). The GRN-associated increase of FLNC was confirmed in the frontal cortex of aged Grn knockout mice starting at 16–18 months of age. Combined quantitative proteomic and bioinformatic analyses of the frontal cortex of FTD patients possessing elevated FLNC levels, identified multiple altered protein factors involved in accelerated aging, neurodegeneration and synaptogenesis.

Our findings further support the involvement of aberrant FLNC expression levels in FTD pathogenesis. Identification of increased FLNC levels in aged Grn mice and impaired pathways related to aging and neurodegeneration, implies a potential role for FLNC in mediating or accelerating the aging process.

**Electronic supplementary material:**

The online version of this article (doi:10.1186/s40478-015-0246-7) contains supplementary material, which is available to authorized users.

## Introduction

Frontotemporal dementia (FTD) is a progressive presenile dementia with degeneration of the frontal and anterior temporal lobes of the brain [1]. The clinical phenotype of FTD patients is heterogeneous and includes cognitive, behavioral and language impairments with a variable co-occurrence of amyotrophic lateral sclerosis (ALS) [2]. Formation of insoluble protein deposits, largely composed of ubiquitinated, hyperphosphorylated TAR DNA-binding protein 43 (TDP-43), defines one of the major pathological subtypes of frontotemporal lobar degeneration (FTLD-TDP) as well as ALS patients, introducing a clinico-pathological continuum of TDP-43 proteinopathies [3–5]. TDP-43 is a multifunctional RNA-binding protein involved in different RNA-related processes including transcription and splicing regulation [6, 7].

Over the past ten years, considerable progress has been made in unraveling the genetic basis of the FTD-ALS continuum. Today, more than 10 genes are linked to FTD-ALS disorders at variable frequencies [8, 9]. A major part of the mutation spectrum described for FTLD-TDP patients is covered by loss-of-function mutations in progranulin (*GRN*) that codes for a multifunctional growth factor with neurotrophic properties in the central nervous system (CNS) [10] and a hexanucleotide repeat expansion mutation in *C9orf72* [11]. Less frequently*,* mutations in TANK-binding kinase 1 (*TBK1*), TAR DNA-binding protein 43 (*TARDBP*), valosin-containing protein (*VCP*), sequestosome 1 (*SQSTM1*) and ubiquilin 2 (*UBQLN2*) can lead to both familial and sporadic forms of FTD and ALS [8].

**Fig. 1:**
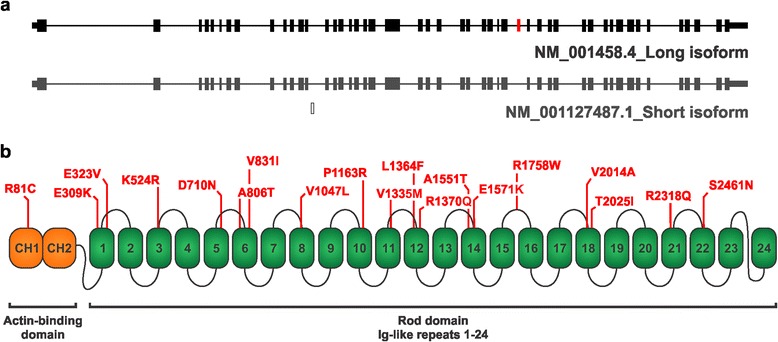
*FLNC* variants identified in patients of the Belgian FTD cohort. **a** Schematic representation of the short and long isoform of *FLNC*, which differ from each other in the presence or absence of one exon (marked in red). **b** 19 *FLNC* variants identified in patients of the Belgian FTD cohort (marked in red) mapped on the domain structures of the *FLNC* gene. Represented variants were absent from 920 control individuals. Immunoglobulin (Ig)-like domains are numbered from 1 to 24. CH1 and CH2: calponin homology domains. Assignment of *FLNC* variants to the corresponding domain structure was based on the UniProt database (http://www.uniprot.org/)

The high prevalence of TDP-43 pathology in FTD and ALS patients suggests that pathways disrupting TDP-43 integrity might be shared between patients with different clinical, pathological and genetic etiologies. In line with the molecular genetic findings, multiple pathways related to RNA-processing, protein aggregation and proteostasis are likely contributing to the multifactorial nature of FTD-ALS disorders [9]. Recently, an unexpected requirement of TDP-43 for muscle maintenance, vessel patterning and perfusion was found upon deletion of the TDP-43 homologues in zebrafish [12]. Several muscle-specific proteins were altered on proteomic analysis of this zebrafish model, underscoring the role of TDP-43 in muscle integrity. The most upregulated protein in TDP-43 knockout zebrafish and in the frontal cortex of FTLD-TDP patients, however, was filamin C (FLNC) [12]. Filamins are evolutionary conserved, multidomain actin-binding proteins involved in the organization of the cytoskeleton and plasma membrane stabilization. Besides cross-linking F-actin filaments, filamins scaffold also a wide range of signaling functions through interactions with more than 90 binding partners including intracellular signaling molecules, transmembrane receptors, and ion channels [13, 14]. The vertebrate filamin family consists of filamin A (FLNA), filamin B (FLNB) and filamin C (FLNC) which share ≈ 70 % homology over the entire protein sequence. Structurally, FLNC is a large homodimer of approximately 290-kDa subunits that consists of an N-terminal actin-binding domain (ABD), composed of two calponin homology domains (CH1 and CH2), followed by 24 Immunoglobulin (Ig)-like repeats [15–17]. Two splice variants have been described for *FLNC* which differ from each other in the presence of exon 31 encoding the hinge region between Ig-like domains 15 and 16 (Fig. 1a). FLNC expression is largely restricted to cardiac and skeletal muscles, although other non-muscular cells, including neuronal cells, express lower but detectable levels of FLNC [18–20]. In line with the expression pattern of FLNC, mutations identified in *FLNC* have been shown to be the underlying cause of different progressive muscular dystrophies, including distal and myofibrillar myopathy (DM and MFM, respectively), and hypertrophic cardiomyopathy (HCM) [21–23].

In the present study, we showed that rare variants (MAF < 1 %) identified in the coding region of *FLNC* are significantly associated with a higher risk of developing FTD. We also performed an in-depth characterization of FLNC transcript and protein levels in FTD patients with different genetic etiologies and found that elevated FLNC levels observed in the frontal cortex of FTLD-TDP patients are mainly associated with the *GRN* p.0(IVS1 + 5G > C) loss-of-function mutation. Validation of this novel association was obtained using a constitutive Grn knockout (Grn^−/−^) mouse model [24]. To appreciate the pathophysiological relevance of increased FLNC levels, we analyzed the frontal cortex of FTD patients with different genetic etiologies for significantly altered gene-specific pathways using combined proteomic and bioinformatic approaches.

## Materials and methods

### Belgian FTD and control cohorts

The FTD patients and control cohorts were ascertained in the framework of the Belgian Neurology (BELNEU) consortium, a multicenter collaboration of dementia expertise centers located in Belgium, covering Flanders, Wallonia and Brussels [25]. Molecular genetic screening of *FLNC* was performed on 529 FTD patients with an average age at onset of 63.8 ± 10.3 years (45.5 % women). Index patients were evaluated and diagnosed according to standard protocols including detailed neurological examination, neuropsychological testing, neuroimaging, biochemical analyses, and electroencephalography (EEG) [26]. Clinical diagnosis of FTD was reached according to the international Lund and Manchester group criteria for FTD [1] and the international consensus criteria by Rascovsky et al. [27] for behavioral variant FTD (bvFTD). Post mortem neuropathological diagnosis of FTLD was available in 33 patients (6.2 %), including 3 FTD-ALS patients, 1 patient with mixed dementia and 25 FTD patients. A positive family history, i.e. at least one first degree relative with a FTD-ALS spectrum disease, was recorded for 30.4 % of the FTD cohort, while 33.8 % had a sporadic form of the disease. Familial history was unknown for 35.2 % of the patients included in this cohort. FTD patients included in this cohort had previously been screened for mutations in known FTD and ALS genes including *MAPT, GRN*, *C9orf72*, *VCP*, *CHMP2B*, *TARDBP*, *FUS*, and *SQSTM1* [9], which revealed 5 *MAPT* mutations (0.9 %), 28 *GRN* mutations (5.3 %), 35 *C9orf72* repeat expansion mutations (6.6 %), 6 *VCP* mutations (1.1 %), 1 *CHMP2B* mutation (0.2 %), 2 *TARDBP* mutations (0.4 %), 3 *FUS* mutations (0.6 %), and 10 *SQSTM1* mutations (1.9 %).

The control cohort consisted of 920 unrelated and age-matched individuals, primarily community-dwelling volunteers or spouses of patients, with an average age at inclusion of 68.0 ± 13.2 years (60.8 % women). Subjective memory complaints, neurologic or psychiatric antecedents and a familial history of neurodegeneration were ruled out by means of an interview. Cognitive screening was performed using the Mini-Mental State Examination (MMSE; cut-off score ≥26) [28] or the Montreal Cognitive Assessment (MoCA) test (cut-off score >25) [29]. The spouses of patients were examined at the Memory Clinic at Middelheim or Hoge Beuken hospitals of the Hospital Network Antwerp, Belgium, and at the Memory Clinic of the hospital Gasthuisberg of the University Hospitals of Leuven, Belgium.

All research participants or their legal guardian provided written informed consent for participation in genetic and clinical studies. Clinical study protocols and informed consent forms for patient ascertainment were approved by the local medical ethics committees of the collaborating neurological centers in Belgium. Genetic study protocols and informed consent forms were approved by the ethics committees of the University Hospital of Antwerp and the University of Antwerp, Belgium.

### *FLNC* sequencing

The coding sequence of *FLNC*, including flanking intron-exon boundaries, was screened in the Belgian FTD and control cohorts using a massive parallel sequencing approach and a customized Multiplex Amplification of Specific Targets for Resequencing (MASTR) assay (Multiplicom; www.multiplicom.com). Exons 46-48 of *FLNC* were not covered in the MASTR assay due to more than 98 % sequence identity with the *FLNC* pseudogene (*pseFLNC*), located 53.6 kb downstream of the functional *FLNC* gene [30]. Primers for multiplex PCR were designed using the mPCR primer design tool (Multiplicom) [31]. Specific target regions were amplified using multiplex PCR, and equimolar pooled amplicons were purified using Agencourt AMPureXP beads (Beckman Coulter). Individual barcodes (Illumina Nextera XT) were incorporated in a universal PCR step prior to sample pooling. Bridge amplification and sequencing of barcoded samples was performed using an Illumina MiSeq platform, with the Illumina v2 reagent kit.

Alignment and mapping of the reads against the human genome reference sequence hg19 were performed with the Burrows-Wheeler Aligner [32]. Variant calling and annotation were performed using GATK (version 2.2) in combination with GenomeComb software [33, 34]. Identified variants were independently validated using direct Sanger sequencing on genomic DNA. FLNC codon numbering was based on GenBank Accession Number NM_001458.4 and amino acid substitutions are numbered according to GenPept Accession Number NP_001449.3 (http://www.ncbi.nlm.nih.gov/).

The potential pathogenecity of patient-only coding variants were predicted using SIFT (http://sift.jcvi.org/) [35], PolyPhen-2 (http://genetics.bwh.harvard.edu/pph2/) [36] and SNAP^2^ (https://www.rostlab.org/services/SNAP/) [37] prediction software tools.

### Human and murine brain tissue

Human frontal cortex (BA10) was snap frozen in liquid nitrogen upon autopsy and stored at −80 °C for subsequent mRNA and protein analyses. FLNC expression levels were analyzed in brain tissue from 23 dementia patients including 1 *FLNC* p.V831I carrier, 7 carriers of a *GRN* loss-of-function mutation [10], 3 carriers of the *VCP* p.R159H mutation [38], 3 carriers of a *C9orf72* repeat expansion [11], 4 patients with FTLD-TDP brain pathology but no mutation in any of the known causal FTD genes, and 1 patient with a mixed Alzheimer’s disease (AD) and FTLD-TDP brain pathology. In addition, we analyzed brain expression levels in 2 patients with AD, one patient with dementia with Lewy Bodies (LBD) and one Down syndrome patient, as well as in twelve age-matched control persons. A detailed overview of the patients with autopsy and neuropathological examination is provided in Table 1. A partial overlap in patients is present with samples included in the FLNC expression studies of Schmid et al. 2012 [26]; i.e. control individuals (*n* = 12), 5 FTD patients (2 *GRN* p.0(IVS1 + 5G > C) carriers, 2 *C9orf72* repeat expansion carriers, 1 FTLD-TDP patient without known genetic cause, 2 AD patients and 1 LBD patient (FTD patient samples used in both studies are indicated with a "symbol a" in Table 1).

**Table 1:** Pathological and clinical characteristics of FTD, AD and DLB patients used in FLNC brain expression studies

DR number	Gender	Age at onset (years)	Age at death (years)	Family History	Clinical diagnosis	Mutation	Pathological diagnosis
DR287.1	F	65	71	F	FTD	*GRN* - p.A89Vfs^a^41	FTLD-TDP type A
DR2.3^a^	F	63	71	F	FTD	*GRN* - p.0(IVS1 + 5G > C)	FTLD-TDP type A
DR8.1	F	62	68	F	FTD	*GRN* - p.0(IVS1 + 5G > C)	FTLD-TDP type A
DR25.5^a^	M	70	73	F	FTD	*GRN* - p.0(IVS1 + 5G > C)	FTLD-TDP type A
DR27.1	F	58	63	F	FTD	*GRN* - p.0(IVS1 + 5G > C)	FTLD-TDP
DR28.1	M	56	62	F	FTD	*GRN* - p.0(IVS1 + 5G > C)	FTLD-TDP type A
DR25.1	F	69	75	F	FTD	*GRN* - p.0(IVS1 + 5G > C)	FTLD-TDP type A
DR40.1	F	44	56	F	FTD	*VCP* - p.R159H	FTLD-TDP type D
DR40.7	M	49	57	F	FTD	*VCP* - p.R159H	FTLD-TDP
DR7.4	M	63	68	F	FTD	*VCP* - p.R159H	FTLD-U
ADR1	M	52	62	F	FTD	*FLNC* - p.V831I	Pick’s disease
DR439.1	M	54	69	F	FTD	*C9Orf72* - G_4_C_2_ expansion	FTLD-TDP type B
DR29.1^a^	F	50	55	F	FTD	*C9Orf72* - G_4_C_2_ expansion	FTLD-UPS
DR14.1^a^	M	56	60	F	FTD	*C9Orf72* - G_4_C_2_ expansion	FTLD-TDP type B
DR386.1	M	72	83	S	MXD	unknown	FTLD-TDP and AD
DR189.1	M	47	50	F	FTD	unknown	FTLD-TDP type B
DR87.1	F	79	88	S	FTD	unknown	FTLD TDP
DR864.1	M	59	62	S	FTD - ALS	unknown	FTLD-TDP type B
DR102.1^a^	F	72	79	S	FTD	unknown	FTLD-TDP
DR246.1^a^	M	62	72	F	DLB	unknown	LBD
DR865.1	M	75	86	U	AD	unknown	AD (III-IV Braak)
DR39.1^a^	F	61	75	F	AD	*APP* - c.-369C/G	AD-CAA
DS1.1^a^	-	-	-	S	Down Syndrome	47XX,+21 or 47XY,+21	AD-CAA

AD *Alzheimer’s disease*, APP *amyloid precursor protein*, CAA *cerebral amyloid angiopathy*, DLB *dementia with Lewy bodies*, UPS *ubiquitin proteasome, system*, DLBD diffuse Lewy body disease, F *familial*; S *sporadic*, U *unknown*, MXD *mixed dementia*, VCP *valosin-containing protein*

Samples labeled with ^a^ were previously analyzed in FLNC expression studies published by Schmid et al. 2012 [12]

Human FLNC expression profiles obtained from FTD patients were validated using a progranulin knockout (Grn^-/-^) mouse model for FTD [24]. Mouse whole brain tissue was harvested at different ages, weighed and cut midsagittal according to a standard protocol [39]. Right hemispheres were snap frozen in liquid nitrogen and stored at −80 °C for subsequent mRNA and protein analysis. Left hemispheres were fixed in 2 % paraformaldehyde (PFA) for 18–20 h and prepared for paraffin embedding.

### RNA extraction for semi-quantitative real-time PCR

Semi-quantitative real-time PCR (qRT-PCR) was performed to quantify expression levels of *FLNC* in the human frontal cortex and mouse right hemisphere. Total RNA was isolated from crunched frozen brain tissue using the RiboPure™ RNA Purification kit followed by a DNase treatment with the TURBO™ DNase kit (both Ambion, Life Technologies). The RNA integrity values (RIN) of patient and control samples ranged between 5 and 8.4. First-strand cDNA was synthesized utilizing the SuperScript® III First-Strand Synthesis System (Life Technologies) with random hexamer primers. qRT-PCR reactions were performed using the Fast SYBR® Green mix and run on an ABI ViiA™ 7 Real-Time PCR System (both Applied Biosystems, Life Technologies). Each sample was measured in duplicate and at least two independent experiments were performed. Quantification of mRNA levels was achieved with glyceraldehyde 3-phosphate dehydrogenase (*GAPDH*) and β-Actin (*ACTB*) as internal control genes. Normalization of the housekeeping genes was performed using geometric averaging of the expression levels, as described by Vandesompele et al. [40]. Primer pairs were designed using Primer Express software (Life Technologies). Primer sequences are available upon request.

### Immunoblotting

Protein extractions of human and murine brains were prepared in modified radioimmuno-precipitation (RIPA) buffer [150 mM NaCl, 0.5 % sodium deoxycholate, 1 % NP-40, 50 mM Tris–HCl; pH 8.0] supplemented with 1 % sodium dodecyl sulfate (SDS), as described previously [41]. Buffers were supplemented with protease and phosphatase inhibitors (1x Complete Protease inhibitor cocktail and 1x PhosSTOP, both Roche). Protein concentrations were determined with a Pierce™ bicinchoninic acid (BCA) colorimetric protein assay kit (Thermo Scientific). Equal amounts of protein were loaded and separated on 3–8 % NuPAGE® Tris-Acetate or 4–12 % NuPAGE® Bis-Tris gels (Life Technologies) and electrotransferred onto a polyvinylidene difluoride membrane (PVDF, Hybond P; Amersham Biosciences). After protein transfer, membranes were blocked in 5 % skimmed milk in phosphate-buffered saline (PBS) containing 0.1 % Tween® 20 (Merck). Membranes were probed with a range of primary antibodies listed in Additional file 1: Table S1. Immunodetection was performed with host-specific secondary antibodies conjugated with horseradish peroxidase (HRP) and the ECL-plus chemiluminescent detection system (Thermo Scientific). Western blot results were visualized using the ImageQuant™ LAS4000 digital imaging system and quantified with ImageQuant™ TL software (GE Healthcare Life Sciences). Quantitative data were normalized to GAPDH expression levels, as described earlier [39].

### Cell culture

Human cervical carcinoma cells (HeLa; CCL-2; ATCC) were grown in Minimum Essential Medium Eagle (MEM, Life Technologies) supplemented with 10 % fetal calf serum (FCS, Life Technologies) and 500 U/500 μg penicillin/streptavidin. Human neuroblastoma (KELLY) cells (Sigma) were grown in Dulbecco’s Modified Eagle’s Medium (DMEM, Life Technologies) supplemented with 10 % FCS, 2 mM L-Glutamine and 500 U/500 μg penicillin/streptavidin. Cells were propagated in a humidified incubator at 37 °C and 5 % CO_2_.

### RNA interference

*GRN* expression was silenced in HeLa and KELLY cells using three independent gene-specific siRNAs (Ambion, Life Technologies). A non-targeting control siRNA with scrambled sequences (Qiagen) and a negative control (only transfection reagent) were included for each siRNA experiment (sequences are listed in Additional file 1: Table S2). Cells were transiently transfected with 20 nM of gene-specific or scrambled siRNA using siLentFect™ transfection reagent (Bio-Rad) according to the manufacturer’s recommendations. Cellular RNA and protein extractions were prepared 48 h post transfection according to standard protocols [41]. Briefly, cells were washed twice in ice-cold PBS and lysed in RIPA 1 % SDS buffer supplemented with protease and phosphatase inhibitors (1x Complete Protease inhibitor cocktail and 1x PhosSTOP, both Roche). Lysates were sonicated on ice, clarified by centrifugation at 14,000 rpm and supernatant was used for subsequent immunoblotting experiments.

### Sample preparation for iTRAQ labeling and mass spectrometry analysis

Human frontal cortex of 5 patients, including 2 FTD carriers of *GRN* p.0(IVS1 + 5G > C) (DR8.1 and DR2.3), 2 FTD carriers of *VCP* p.R159H (DR40.1 and DR40.7), 1 *FLNC* p.V831I carrier (ADR1) (Table 1), and of 3 control individuals, were extracted using the Qproteome Cell Compartment kit (Qiagen). Proteins extracted from the cytoplasmic compartment using Qproteome were used for subsequent mass spectrometry analyses. Following acetone precipitation of the cytoplasmic protein extracts, protein lysate concentrations were measured using the *RC DC*^*TM*^ protein assay (Bio-Rad Laboratories Inc.). A total protein concentration of 100 μg from each sample was reduced with tris(2-carboxyethyl)phosphine (TCEP) and cysteine residues were blocked using the cysteine-alkylation reagent methyl methanethiosulfonate (MMTS). Next, samples were digested with sequencing-grade trypsin (Promega) and peptides were used for isobaric iTRAQ-8plex (isobaric mass-tag labeling for relative and absolute quantification; iTRAQ [42]) mass tag labeling, i.e. 113, 114, 115, 116, 117, 118, 119 or 121 labels, according to the manufacturer’s protocol (Applied Biosystems). Controls and patients samples were labeled for 1 h at room temperature with isobaric reagents as follows: 3 control individuals (labels 113-115); 2 *GRN* p.0(IVS1 + 5G > C) carriers (labels 117-118), 2 *VCP* p.R159H carriers (labels 119-121) and a *FLNC* p.V831I carrier (label 116). Following labeling, the tagged peptides were pooled in equal volume ratios into one sample mix.

Labeled peptides were separated and resolved in two dimensions, first by strong cation exchange (SCX) chromatography followed by nano-reversed phase (RP, C18) chromatography on a 2D nano liquid chromatography (LC) system (Ultimate 3000 RSL, Dionex, Thermo Scientific). The nano-LC system was connected online to a Thermo Fisher Q Exactive™ Plus Orbitrap Mass Spectrometer (MS) system (Thermo Scientific).

Data-dependent acquisition (DDA) was obtained with a full MS scan and a subsequent data dependent MS2 (ddMS2) scan. The full MS scan had a resolving power of 70,000 and a scan range from 350 to 1500 m/z. The top 10 precursor ions were selected for ddMS2 higher-energy collisional dissociation (HCD) fragmentation with a resolution of 17,500.

Data analysis, including identification and quantification of labeled peptides, was performed using the Proteome Discoverer software (v2.0, Thermo Scientific). Protein identification was performed using the most recently updated human UniProt/SwissProt database. Raw peptide identification was done using workflow settings for the Sequest HT search engine including a 10 ppm precursor mass tolerance, 0.02 Da fragment mass tolerance, and tolerance of maximum two missed trypsin cleavage sites. Only peptide spectrum matches (PSM) that contained all eight reporter ion channels were considered for quantification and reporter intensities were exported for further analysis.

### Bioinformatic analyses of mass spectrometry data

Proteomic datasets generated from the Proteome Discoverer v2.0 software were analyzed using a multidimensional bioinformatic approach. The primary ratiometric iTRAQ data was processed and normalized by performing a log_2_ transformation on the signal ratios retrieved from the quantitative MS. Proteins demonstrating significant deviation limits outside plus or minus 2 standard deviations (SD; 95 % confidence limits) from the mean protein expression levels (based on normalized background analysis) were used for further analysis.

To facilitate the specific separation of complex datasets, and more specifically the significantly-regulated proteins, originating from either the *FLNC*, *GRN* or *VCP* mutation carriers (compared to control individuals), we employed the novel Venn diagram platform, VennPlex [43]. Our primary analyses were focused on significantly-regulated protein subsets containing proteins common to *FLNC*, *GRN*, and *VCP* carriers (FTD-common), but also protein subsets specific and unique to *FLNC*, *GRN* or *VCP* carriers. Classical functional annotation of these protein subsets was performed with Gene Ontology annotation (GO; http://geneontology.org/) and Kyoto Encyclopedia of Genes and Genomes pathway analysis (KEGG; http://www.genome.jp/kegg/) using the NIH DAVID Bioinformatics Resources 6.7 suite [44]. For both GO term annotation (biological process only) and KEGG pathway analyses we employed a cut-off of at least two proteins needing to be present to fully populate a particular GO term group or KEGG pathway with a probability of enrichment value of *P* ≤0.05. A hybrid score was employed to generate a single index for a specifically enriched GO term group or KEGG pathway. Hybrid scores are generated by the multiplication of the negative log_10_ of enrichment probability with the enrichment factor and the number of proteins populating the specific GO term group or KEGG pathway.

Biomedical natural language processing (NLP) text analysis was performed on the common and unique significantly-regulated protein datasets using T*extrous!* [45, 46]. *Textrous!* utilizes NLP techniques, including latent semantic indexing (LSI), sentence splitting, word tokenization, parts-of-speech tagging, and noun-phrase chunking, to mine the MEDLINE-database (NCBI, http://www.ncbi.nlm.nih.gov/), PubMed Central articles (NCBI, http://www.ncbi.nlm.nih.gov/), the Online Mendelian Inheritance in Man catalog (OMIM®, http://www.omim.org/), and the Mammalian Phenotype Browser obtained from The Jackson Laboratory (http://www.informatics.jax.org/searches/MP_form.shtml). *Textrous!* has the ability to generate output data even with very small input datasets for the selection, ranking, clustering, and visualization of English words obtained from the input user data. Significant data word clouds were created from *Textrous!*-based semantic noun and noun-phrase outputs using the web-based application Wordle (http://www.wordle.net/) [45]. The text size of the word clouds is directly proportional to the input word frequency. Analyses of word frequencies from *Textrous!* noun-phrase outputs were made using WriteWords (http://www.writewords.org.uk/word_count.asp).

LSI analysis was performed using GeneIndexer (Computable Genomix), as described previously [47, 48]. In brief, GeneIndexer correlates the strength of association, using LSI, between specific proteins in a dataset with user-defined interrogation text terms. GeneIndexer employs a comprehensive human or murine scientific text database of ≥2 × 10^6^ scientific abstracts to perform this text-protein correlation analysis. The possible LSI cosine similarity correlation scores for a gene to be associated with an input interrogation term range from 0 to 1, with the stronger correlation scores approaching 1. A correlation score of ≥0.1 indicates at least an implicit correlation, between the specific gene and the user-defined input interrogation term.

### Statistical analyses

We determined rare, low frequency and frequent variants as genetic variants with a minor allele frequency (MAF) below 1 %; between 1-5 % and above 5 %, respectively. Rare variant gene burden analysis was performed by collapsing rare alleles and comparing the overall frequency of rare variant alleles between patients and control groups using chi-squared (*χ*^2^) statistics. For two controls, more than one FLNC variant was identified in the CDS and these variants were counted as one mutated allele as no relatives were available for phase determination of the variants. The odds ratio (OR) and 95 % confidence intervals (CI) were calculated as well. A two-sided p-value *P* <0.05 was considered as significant.

Expression studies were performed in duplicate and repeated at least two times with results reported as mean ± standard deviation (SD). *P* values for description of statistical significance of differences were calculated by Mann–Whitney U testing using the GraphPad Prism 5 Software. Values were considered to be significant if *P < 0.05, **P < 0.01 or ***P <0.001.

## Results

### Identification of rare *FLNC* variants in Belgian FTD patients

We sequenced the coding sequence (CDS) of *FLNC* in the Belgian FTD (*n* = 529) and control (*n* = 920) cohorts and identified a total of 68 different genetic variants that affected the coding sequence of *FLNC.* We observed 19 missense variants (MAF <1 %) in 21 patients, which were absent from control individuals (Fig. 1b, Table 2). The clinical diagnosis of the *FLNC* carriers was predominantly FTD, except for the 2 patients with p.D710N and p.T2025I, who were diagnosed with FTD-ALS and corticobasal syndrome (CBS) (Table 2). Eight carriers had a positive family history of disease, but their families lacked information preventing co-segregation analysis with disease. The patient-only variants were found across the actin binding domain and different Immunoglobin (Ig)-like domains of FLNC without indications of clustering in specific functional domains (Fig. 1b, Table 2). Comparative genomics analysis indicated that the majority of *FLNC* variants predicted substitution of evolutionary conserved amino acid residues in FLNC (Additional file 2: Figure S1). For 5 *FLNC* variant carriers with FTD, another genetic causal or risk variant had previously been identified (Table 2), 2 with a *C9orf72* repeat expansion, 2 with a *GRN* loss-of-function mutation and 1 with the *TREM2* p.R47H risk allele (Table 2).

**Table 2:** Rare *FLNC* missense variations identified in Belgian FTD patients

Patient	Variant	Functional domain	Gender	Clinical diagnosis	Sub-diagnosis	Family History	Age at onset (years)	dbSNP	Mutation in known gene
DR554.1	p.R81C	CH1	F	FTD	bvFTD	F	56	-	*GRN* p.W304
DR1221.1	p.E309K	Ig 1	M	FTD	PNFA	S	45	-	
DR1222.1	p.E323V	Ig 1	M	FTD	bvFTD	S	71	-	
DR659.1	p.K524R	Ig 3	M	FTD	bvFTD	F	38	-	*C9orf72* G_4_C_2_ exp.
DR454.1	p.D710N	Ig 5	F	FTD-ALS	FTD - ALS	F	69	rs370035829	*C9orf72* G_4_C_2_ exp.
DR1223.1	p.A806T	Ig 6	F	FTD	PSP	F	63	-	
ADR1	p.V831I	Ig 6	M	FTD	bvFTD	F	52	-	
DR1224.1	p.V1047L	Ig 8	F	FTD	-	S	57	-	
AD1485.1	p.P1163R	Ig 10	M	FTD	bvFTD	F	76	-	
AD1430.1	p.V1335M	Ig 11	M	FTD	bvFTD	S	47	rs368220468	
DR1225.1	p.L1364F	Ig 12	F	FTD	PSP	U	83	-	
AD1788.1	p.R1370Q	Ig 12	M	FTD	-	S	68	-	
DR867.1	p.A1551T	Ig 14	M	FTD	bvFTD	F	77	-	
DR559.1	p.E1571K	Ig 14	F	FTD	bvFTD	F	60	-	*TREM2* p.R47H
DR1226.1	p.E1571K	Ig 14	M	FTD	SD	S	75	-	
DR355.1	p.R1758W	Linker Ig 15–16	F	FTD	bvFTD	S	75	rs369187211	
DR825.1	p.V2014A	Ig 18	F	FTD	PNFA	S	66	-	
AD1462.1	p.T2025I	Ig 18	F	FTD	PPA + EPS	S	62	-	*GRN* p.0(IVS1 + 5G > C)
DR1227.1	p.T2025I	Ig 18	F	CBS	CBS	U	70	-	
DR868.1	p.R2318Q	Ig 21	M	FTD	PNFA	S	71	-	
AD1321.1	p.S2461N	Ig 22	F	FTD	-	U	63	-	

bvFTD *behavioral variant frontotemporal dementia*, CBD *corticobasal degeneration*, CBS *corticobasal syndrome*, dbSNP *Single Nucleotide Polymorphism database*, EPS *extrapyramidal syndromes*, exp. *expansion*, F *familial*, GRN *progranulin*, PNFA *progressive nonfluent aphasia*, PPA *primary progressive aphasia*, PSP *progressive supranuclear palsy*, S *sporadic*, TREM2 *Triggering receptor expressed on myeloid cells 2*, U *family history undocumented*

In addition to patient-only variants, we identified another 15 missense *FLNC* variants that were present in both FTD patients and control individuals. Except for p.R1241C and p.R1567Q (MAF >1 % and >5 %), the other 13 were rare (MAF <1 %) (Additional file 1: Table S3a and b). We also identified 34 missense variants present only in control individuals (MAF <1 %) (Additional file 1: Table S4). Three different prediction software programs, i.e. SIFT, Polyphen-2 and SNAP^2^, were applied to estimate the predicted pathogenicity of variants identified in FTD patients and/or controls. However, the vast majority of *FLNC* variants found in patients and/or controls were predicted to have non-neutral effects (Additional file 1: Table S5a-c).

Two control individuals were carrying 2 and 3 different missense variants in *FLNC*. Interestingly, the p.A2430V variant found in one control individual was previously described in patients with hypertrophic cardiomyopathy (HCM) [21].

### Rare variant gene burden analysis in the Belgian FTD cohort

We performed a gene burden analysis collapsing all rare variants with an MAF <1 % across the whole protein and comparing the overall frequency of rare variant alleles between patients and controls using *χ*^2^ statistics. Considering all rare variants observed in patients, we obtained an overall cumulative frequency of 11.3 % (60/529) compared to 7.9 % in control individuals (73/920). The increased frequency in patients was significant with an odds ratio of 1.46 (chi-squared (*χ*^2^) test, *P* = 0.0349; 95 % confidence interval (CI) = 1.03–2.07).

### Increase of FLNC in FTLD-TDP patients is strongly linked to GRN haploinsufficiency

**Fig. 2:**
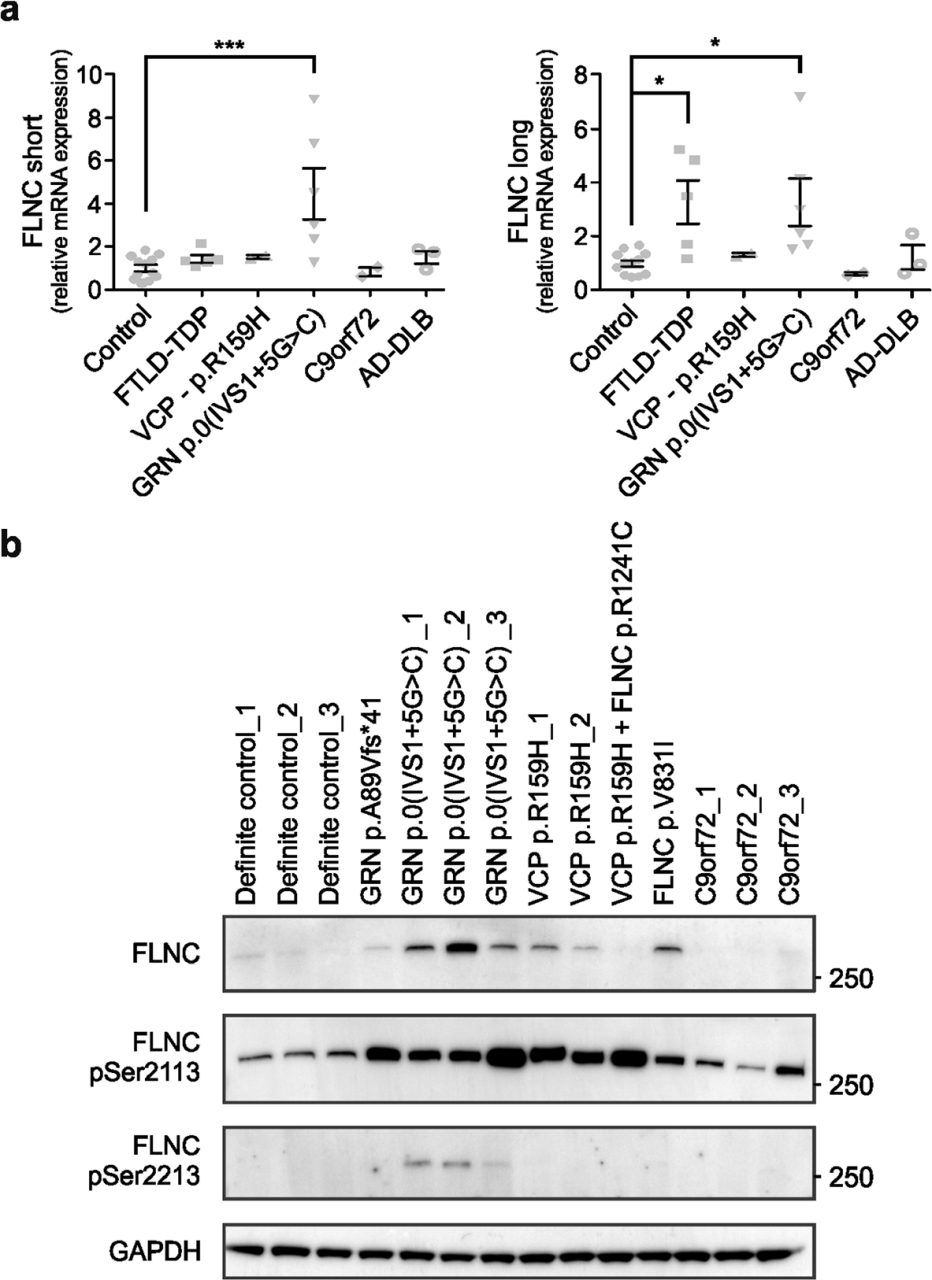
Analysis of FLNC expression in FTD patients at transcript and protein level. **a** qRT-PCR analysis showed significantly increased levels of both the short and long isoform of *FLNC* in the frontal cortex of FTLD-TDP patients with GRN haploinsufficiency. The long isoform was also significantly increased in FTLD-TDP patients without a known mutation in causal FTD-ALS genes. **b** Increased expression levels of *FLNC* were confirmed on protein level using immunoblot analysis. In contrast to transcript levels, FLNC was also upregulated in *VCP* and *FLNC* variation carriers. Elevated phosphorylated FLNC levels at serines 2113 and 2213 (pSer^2113^ and pSer^2213^) were identified to a variable extent in *GRN* and *VCP* mutation carriers compared to controls. *P < 0.05; ***P < 0.001

In the initial report, published by Schmid et al. [12], the expression levels of both the short and long isoforms of human *FLNC* were found to be elevated in the frontal cortex of FTLD-TDP patients, compared to neurologically healthy control individuals and AD patients [12]. We analyzed FLNC expression levels in the frontal cortex (BA10) of FTLD-TDP patients carrying a pathological mutation in *VCP*, *GRN* or *C9orf72* (Table 1), and of FTLD-TDP patients without a known genetic cause (nonmutation FTLD-TDP patients). Compared to transcript levels measured in the frontal cortex of control individuals and of AD or DLB patients, both the short and long isoform of *FLNC* were elevated up to 8.9 and 7.2 times in *GRN* p.0(IVS1 + 5G > C) carriers (Fig. 2a). The long isoform of FLNC was also increased in nonmutation FTLD-TDP patients (Fig. 2a). No altered expression patterns were observed in patients with the *VCP* p.R159H or *C9orf72* repeat expansion mutation.

Western blot analysis, using an antibody raised against the carboxyl terminus of human FLNC confirmed the elevated expression of FLNC in *GRN* p.0(IVS1 + 5G > C) patients compared to age-matched control individuals (Fig. 2b). No notable increase could be observed for the p.A89Vfs*41 frameshift mutation in FLNC expression. In contrast to the transcript data, FLNC protein levels were also elevated to a lower extent in FTD patients carrying the *VCP* p.R159H mutation. No increased FLNC levels were observed in a *VCP* carrier (DR7.4) that also carried the *FLNC* low frequency variant p.R1241C (Additional file 1: Table S3b). Interestingly, analysis of the *FLNC* p.V831I carrier showed an increase in FLNC levels compared to control individuals (Fig. 2b). Absence of elevated FLNC levels in brains of AD patients was in line with the transcript data and the previous data published by Schmid et al. [12] (Fig. 2a and Additional file 2: Figure S2a). As the strongest alterations in FLNC expression were observed in FTD patients with GRN haploinsufficiency, we verified whether reduced GRN levels in vitro could confirm the FLNC increase seen in patients. No significant changes, however, could be observed in FLNC expression levels upon GRN knockdown in both HeLa or KELLY cells (Additional file 2: Figure S3a-c).

Besides its anchoring and crosslinking functions, FLNC is known to scaffold a wide range of signaling pathways through interactions with signal transduction molecules, receptors and ion channels. FLNC is phosphorylated by protein kinase B-α at serine 2213 (pSer^2213^) and harbors also a potential cAMP-dependent protein kinase A (PKA) phosphorylation site at serine 2113 (pSer^2113^) [49]. Western blot analysis of the FLNC phosphorylation status at these reported residues demonstrated a considerable increase in pSer^2113^ levels in *GRN* loss-of-function mutation carriers. In contrast to total FLNC levels, *VCP* mutation carriers also showed a strong increased phosphorylation of FLNC at position pSer^2113^. Surprisingly, the *FLNC* p.V831I showed a lower degree of pSer^2113^levels compared to the low frequency variant (p.R1241C) carrier (Fig. 2b). Upregulation of phosphorylation of FLNC pSer^2213^ could only be detected in *GRN* p.0(IVS1 + 5G > C) carriers and was not present in controls or other FTD-related mutation carriers (Fig. 2b).

### Elevated *Flnc* expression levels are confirmed in a progranulin knockout mouse model

**Fig. 3:**
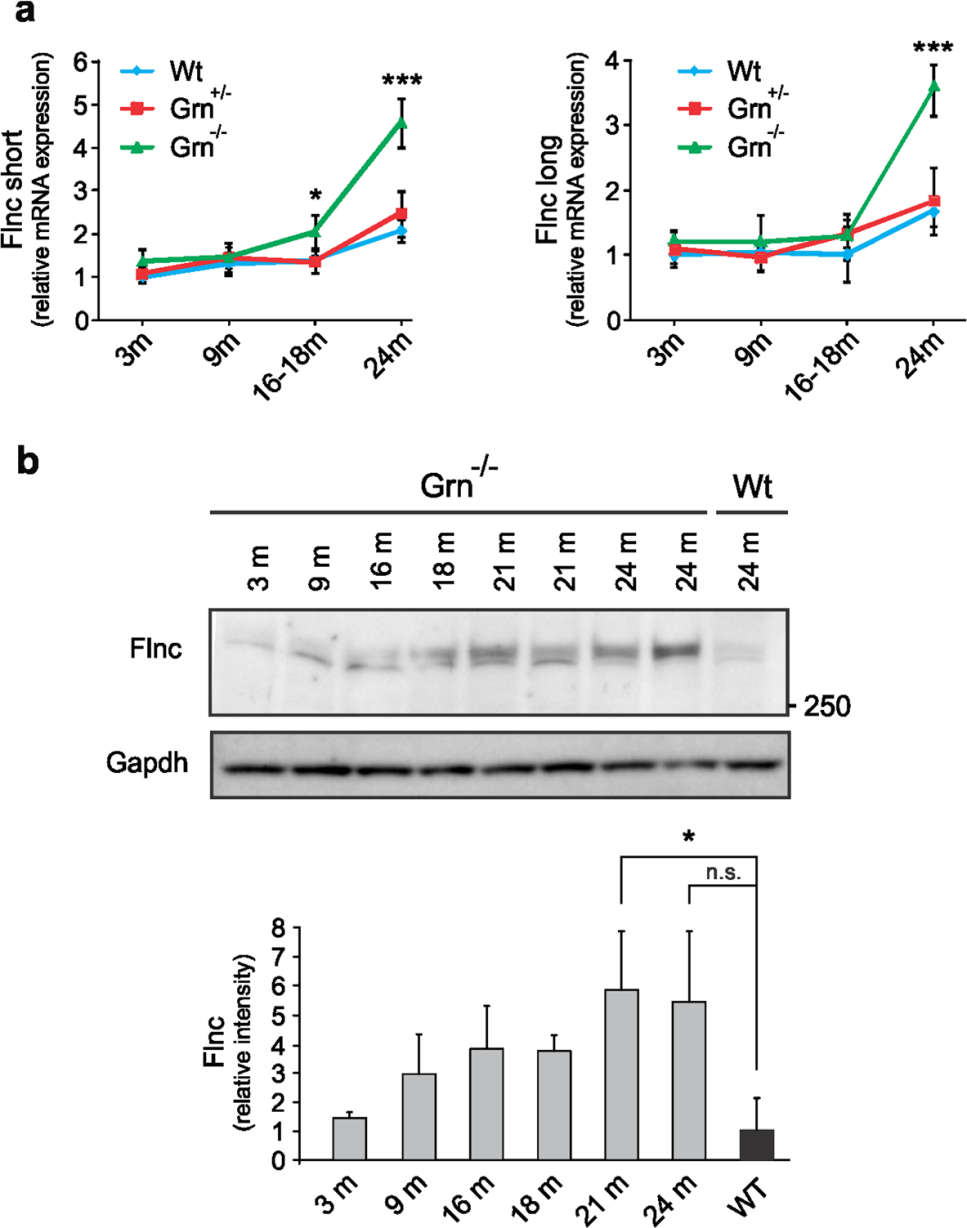
Analysis of Flnc expression in Grn knockout mice at transcript and protein level. qRT-PCR analysis of the long and short isoform of *Flnc* (**a**) measured in progranulin knockout mice of different ages. We analyzed the expression levels of both long and short isoforms of mouse *Flnc* in heterozygous Grn^+/-^ and homozygous Grn^-/-^ mice and wild-type (Wt) animals of 3 months (*n* = 4), 9 months (*n* = 4), 16-18 months (*n* = 6) and 24 months (*n* = 5) of age. **b** Increased *FlnC* expression levels were confirmed on protein level using quantitative immunoblot analysis. Two protein bands are detected around the height of mouse FLNC using the Kinasource AB152 anti FLNC antibody. The upper band is FLNC specific as determined by Western blotting using lysates from FlnC knockout mice (data not shown). The lower band is therefore considered as an aspecific protein band. *P < 0.05; ***P < 0.001, *n.s. not significant*

We aimed at validating the altered transcript profile present in FTLD-TDP patients with GRN haploinsufficiency in progranulin (*Grn*) knockout mice [24]. We analyzed expression levels of both long and short isoforms of mouse *Flnc* in heterozygous Grn^+/-^ and homozygous Grn^-/-^ transgenic mice compared to wild-type (Wt) animals at 3 months, 9 months, 16–18 months and 24 months of age. Consistent with the data obtained for FTLD-TDP patients with GRN haploinsufficiency, transcript levels of both *Flnc* isoforms increased significantly in an age-dependent manner in Grn^-/-^ brains starting from an age of 16–18 months and increasing to a fivefold at 24 months of age (*P* <0.0001) (Fig. 3a). No changes in *Flnc* isoform expression were observed between heterozygous Grn^+/-^ and Wt mice up to an age of 24 months despite a small age-related tendency to increase in relative values. Utilizing a mouse Flnc-specific antibody, Flnc protein levels were found to progressively increase with age and the strongest increase was detected at an age of 21 months in Grn^-/-^ mice which is comparable to the transcript data (Fig. 3b).

Further in line with the human data is that Flnc levels remained unchanged in two mouse models of AD, including Tg2576 mice overexpressing the Swedish *APP* variant p.KM670/671NL (Taconic Farms Inc.) or T8B7 mice expressing the *PSEN1* p.G384A mutation (Additional file 2: Figure S2b) [50]. Due to the high sequence homology between *Flnc* and other members of the filamin family, we also investigated the expression levels of filamin A (*Flna*, ≈72 % homology) and filamin B (*Flnb*, ≈70 % homology) in our Grn^-/-^ mouse model. No significant alterations were identified for both *Flna* and *Flnb* transcripts at end-stage Grn^-/-^ mice compared to Wt mice (Additional file 2: Figure S2c-d), suggesting that the identified alterations are Flnc specific. Expression of other FTD-related genes, e.g. *Tdp-43*, *Vcp* and *C9orf72* in Grn^-/-^ mice remained unaltered as well (Additional file 2: Figure S2e-g).

### Proteomic investigation of frontal cortex of FTD patients with increased FLNC levels using iTRAQ

**Fig. 4:**
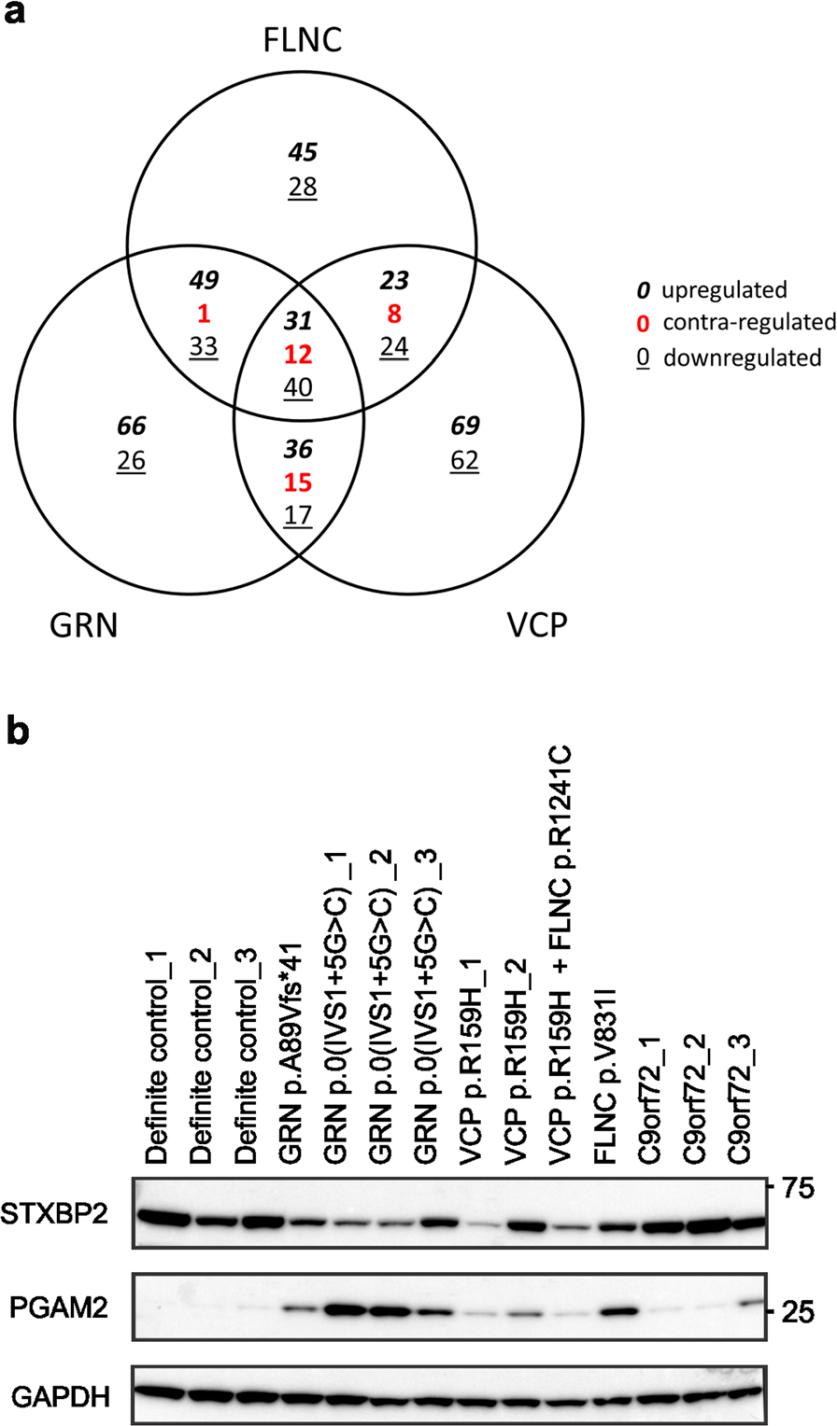
Visualization and validation of FLNC proteomics datasets on FTD patients. (**a**) Three-set Venn diagram separation of the significant protein datasets obtained from proteomic analysis of FTD patients with increased FLNC levels, i.e. *FLNC* p.V831I, *GRN* p.0(IVS1 + 5G > C) and *VCP* p.R159H carriers. Proteins depicted in the Venn diagrams are either up- (italic), down-(underlined) or contra-regulated (red) when compared to expression levels of control individuals (*n* = 3). (**b**) Among the core of most up- or downregulated proteins, phosphoglycerate mutase 2 (PGAM2) and syntaxin binding protein 2 (STXBP2) were validated using Western blot analysis

To investigate the post-genomic sequelae of the human FLNC phenotype we performed an exploratory quantitative proteomic study of the frontal cortex (BA10) from patients carrying the *FLNC* p.V831I variant, the *GRN* p.0(IVS1 + 5G > C) mutation and *VCP* p.R159H mutation compared to control individuals. *C9orf72* repeat expansion carriers were not included due to the absence of a detectable FLNC increase. We found that 294, 326 and 337 proteins were significantly and differentially regulated for FLNC (Additional file 1: Table S6), GRN (Additional file 1: Table S7) and VCP (Additional file 1: Table S8) patients, respectively. Venn diagram separation of these significant protein datasets demonstrated the presence of a core set of 71 common and coherently regulated (with respect to elevated or reduced expression) proteins across all genomic FTD etiologies (Fig. 4a, Additional file 1: Table S9). We validated phosphoglycerate mutase 2 (PGAM2) and syntaxin binding protein 2 (STXBP2) via Western blot analysis (Fig. 4b), which closely paralleled the quantitative mass spectrometry data (exemplary iTRAQ reporter ion analysis for both proteins is represented in Additional file 2: Figure S4a,b). We also explored the nature of the proteins unique to each FTD paradigm, i.e. specific to the *FLNC* (p.V831I), *GRN* (p.0(IVS1 + 5G > C)) or *VCP* (p.R159H) patients (Additional file 1: Table S10-12).

### Differential bioinformatic interpretation of quantitative proteomic analyses

**Fig. 5:**
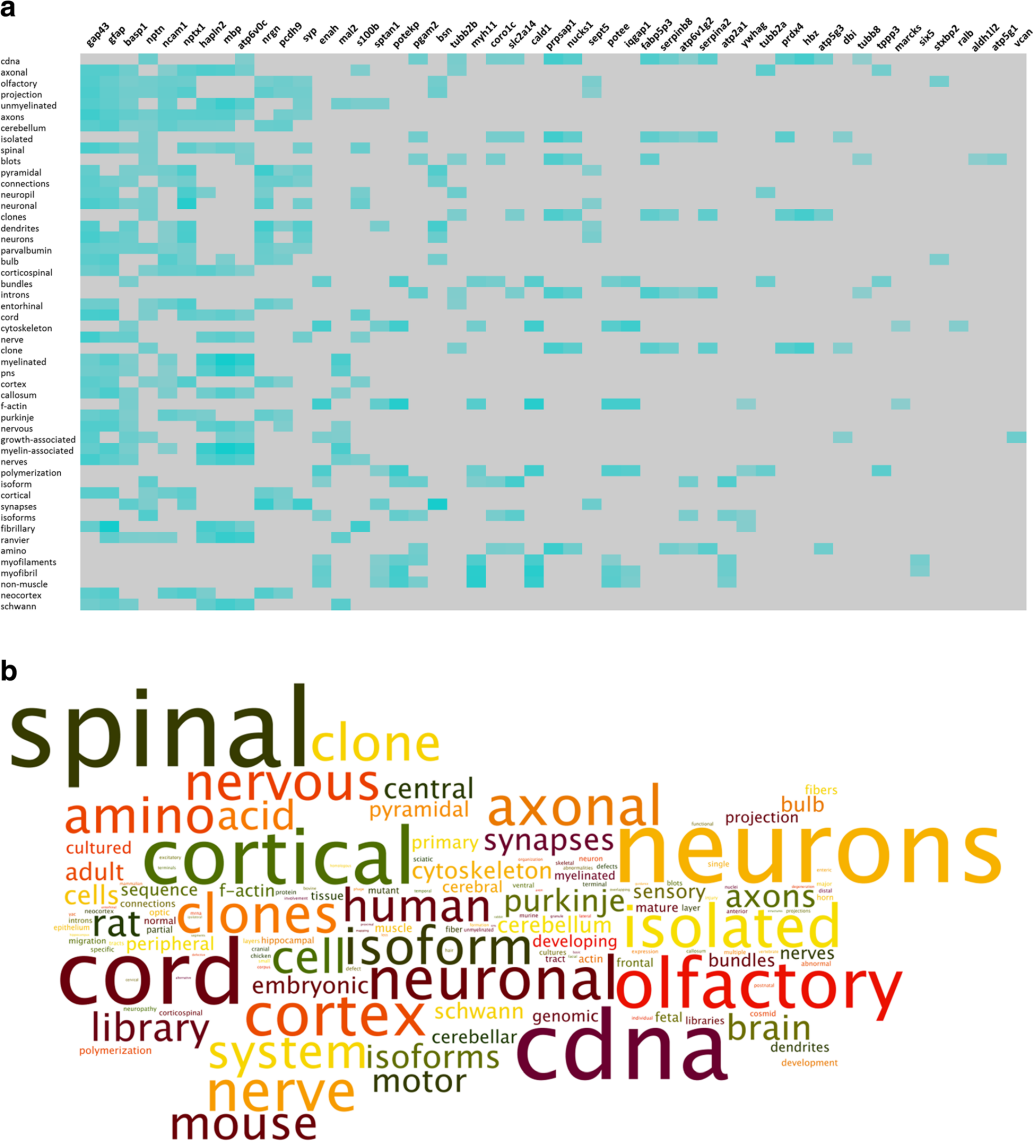
Representation of FTD-common protein dataset using Textrous! and word clouds. (a) Analysis of the FTD-common protein dataset using Textrous! natural language processing (NLP). Strongest correlations between words (vertical) and proteins (horizontal) are presented in a Textrous! heat map. Teal-colored blocks indicate strongly-associated geneword interactions in an intensity-sensitive manner; grey blocks indicate no significant interaction. (b) Word clouds obtained from Wordle (http://www.wordle.net/) analyzing the Textrous! output data common to all FTD patients

To create a gestalt biomedical appreciation of the differential expression datasets, we employed *Textrous!* natural language processing (NLP) based analysis to generate a focussed semantic interpretation of the common FTD dataset and the FLNC-, GRN- and VCP-unique datasets. Using the *Textrous!* individual processing module we found for the FTD-common protein dataset the strongest correlations between the words ‘*dendrites*’, ‘*growth-associated’*, ‘*cytoskeleton*’, ‘*polymerization*’, and ‘*synapses*’ and following proteins: growth associated protein 43/neuromodulin (GAP43), glial fibrillary acidic protein (GFAP), brain acid soluble protein 1 (BASP1), neuroplastin (NPTN) and neuronal pentraxin-1 (NPTX1) (Fig. 5a). To effectively condense the terms involved in the individual processing matrix we extracted all the semantically-associated nouns and noun-phrases from this output to generate a higher-order word cloud (Fig. 5b, Additional file 1: Table S13a), reinforcing the strong cytoskeletal/dendritic/synaptic focus of the FTD-common dataset.

**Fig. 6:**
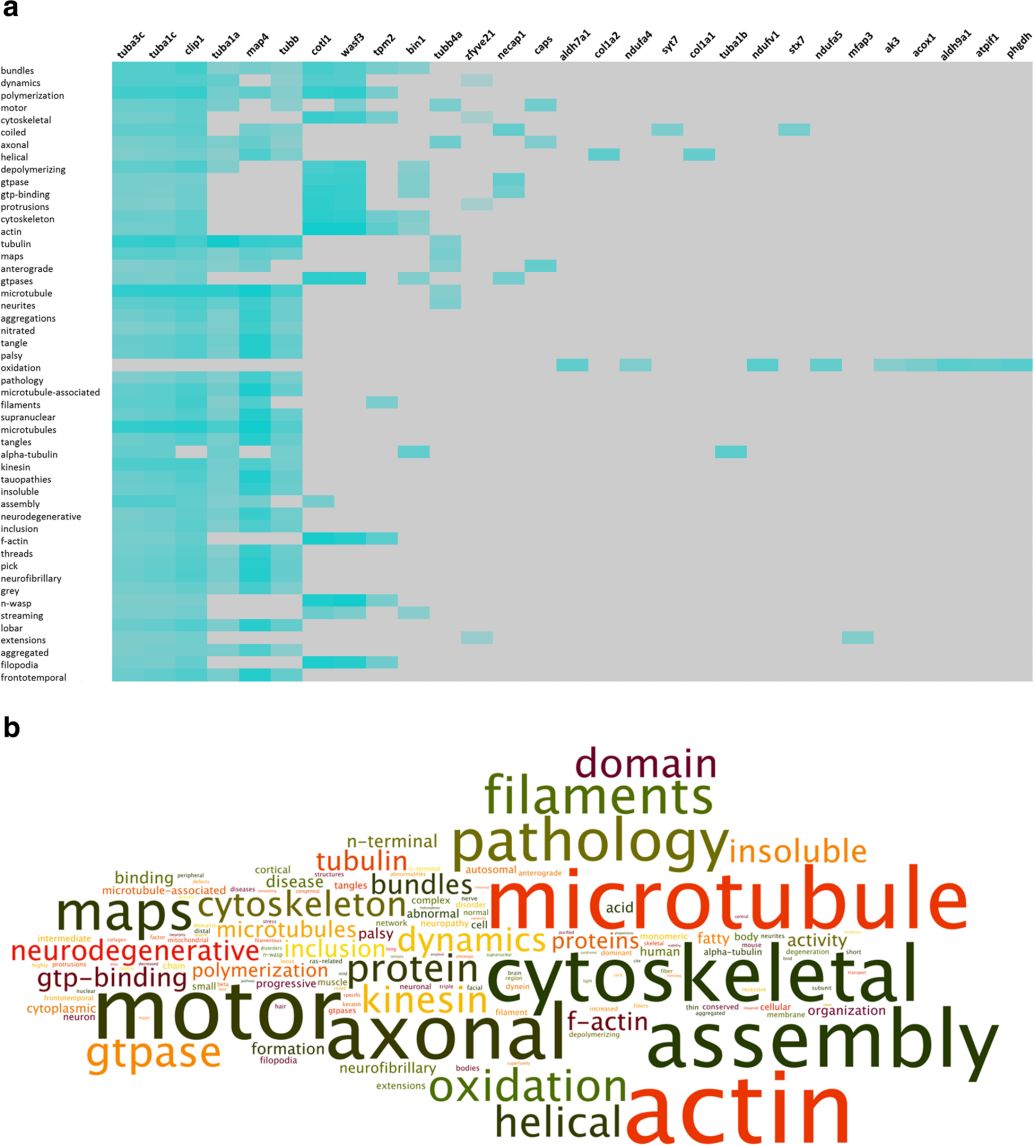
Representation of the FLNC p.V831I unique protein dataset using Textrous! and word clouds. (a) Analysis of the FLNC p.V831I unique protein dataset using Textrous! natural language processing (NLP). Strongest correlations between words (vertical) and proteins (horizontal) are presented in a Textrous! heat map. Teal-colored blocks indicate stronglyassociated gene-word interactions in an intensity-sensitive manner; grey blocks indicate no significant interaction (b) Word clouds obtained from Wordle (http://www.wordle.net/) analyzing the Textrous! output data unique for the FLNC p.V831I variant

We next focussed on a similar NLP-based interpretation of the FLNC-specific protein dataset. Repeating the *Textrous!* NLP analysis for the *FLNC* p.V831I variant (Fig. 6a), as with the FTD-common dataset, we found strong correlations between the words ‘*neuronal-Wiskott–Aldrich Syndrome protein* (*N-wasp)’*, ‘*cytoskeleton*’, ‘*neurites*’, ‘*microtubules*’ and ‘*filopodia*’ and following proteins: CAP-GLY domain containing linker protein 1 (CLIP1), Wiskott-Aldrich syndrome protein family member 3 (WASF3), Myc box-dependent-interacting protein 1 (BIN1) and microtubule-associated protein 4 (MAP4). Interestingly, and in contrast to the FTD-common dataset interpretation, a strong neurodegenerative focus was also present in the FLNC dataset interpretation, i.e. semantic association with multiple disease-related words including ‘*aggregations*’, ‘*oxidation*’, ‘*tangles*’, ‘*tauopathies*’, ‘*neurodegenerative*’, ‘*inclusion*’ and ‘*frontotemporal*’. This dual functionality of the FLNC-unique dataset, i.e. cytoskeleton/neurodegeneration was also strongly evident from the extracted higher-order word cloud (Fig. 6b, Additional file 1: Table S13b). Hence the FLNC proteomic phenotype appears closely focused on neuroskeletal mechanisms tightly linked with neurodegenerative activity.

Using an identical NLP-based informatic approach for the GRN- (Additional file 2: Figure S5a, b) and VCP-unique (Additional file 2: Figure S6a, b) datasets we found that the GRN dataset was focussed upon ‘*transporter*’, ‘*mitochondrial*’, ‘*oxidation*’ and ‘*cellular motility’* activity while in contrast the VCP-unique dataset was more focussed upon neuronal ‘*synaptic*’, ‘*vesicle*’, ‘*transmission*’ and ‘*plasticity*’ functions (Additional file 1: Table S13c-d). A general overview of the top 10 highest frequency words for each ‘higher-order’ word cloud, both FTD-common and the individual unique datasets, are outlined together in Additional file 1: Table S13a-d.

**Fig. 7:**
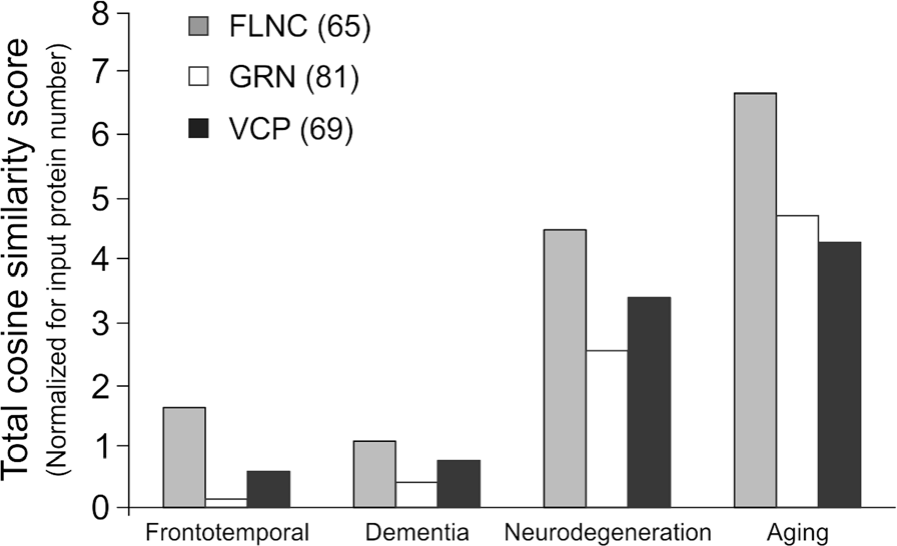
Total cosine similarity scores of the FLNC proteomics datasets on FTD patients. Summation of the total cosine similarity scores for the input interrogator terms showed that the FLNC-unique protein dataset demonstrated the strongest semantic correlation to multiple factors associated with FTD compared to the GRN or VCP-datasets. Factors included in the analysis comprise frontotemporal, dementia, neurodegeneration and aging. Calculations take into account the relative sizes of input datasets

Overall, it is evident that for the FLNC-, GRN- and VCP-specific paradigms a specific and idiosyncratic post-genomic FTD-related functionality is present. As we found that the FLNC-unique protein dataset was strongly associated with a neurodegenerative/oxidative damage signature we attempted to independently quantify this using an orthogonal informatic approach to *Textrous!*. Hence we sought to evaluate the semantic correlation between user-defined words associated with neurodegeneration, dementia and aging and proteins from the FLNC-, GRN- or VCP-specific datasets. This was performed using the LSI-based GeneIndexer application. Through summation of the total cosine similarity scores for the input interrogator word terms and taking into account the relative sizes of input datasets, we found that multiple factors in the FLNC-specific protein dataset (Fig. 7) demonstrated the strongest semantic correlations to multiple terms associated with FTD (Additional file 1: Table S14-16). Therefore it appears that, for the FLNC-FTD patients, both protein and bioinformatic associations demonstrate a relatively unique and profound connection to FTLD pathology.

## Discussion

Investigating the pathogenicity of TDP-43 deletion in zebrafish by Schmid et al. [12] demonstrated a potential role for muscle-specific proteins in the disease mechanism underlying FTD-ALS disorders, with FLNC being the most up-regulated protein. A potential link between the loss of TDP-43 in zebrafish, and the concomitant altered expression of FLNC, and the human disease was provided by the observation of elevated FLNC levels in the frontal cortex of FTLD-TDP patients. To further explore the potential involvement of FLNC in FTLD pathogenesis, we consequently screened the Belgian FTD patient and control cohorts for the presence of *FLNC* variants associated with FTD and determined the genetic etiology underlying the elevated *FLNC* expression levels in FTLD-TDP patients as observed by Schmid et al. [12]. Molecular genetic screening of the coding sequence of *FLNC* resulted in the identification of 68 missense variants, of which 19 were missense variants identified in 21 FTD patients only. All variants reported in this study are rare (MAF <1 %), except the p.R1241C and p.R1567Q missense variants which were low frequency (1 % < MAF <5 %) and frequent (MAF >5 %) variants, respectively. When considering all rare variants, indifferent if they were present or not in control individuals, FTD patients demonstrated a significant enrichment of rare variants (cumulative variant frequency of 11.3 %) when compared to control individuals (cumulative variant frequency of 7.9 %) (*P* = 0.0349; OR = 1.46, 95 % CI = 1.03–2.07).

Formerly, mutations in *FLNC* were described as an underlying cause of two distinct types of myopathy, known as myofibrillar and distal myopathy (MFM and DM) [17]. These myopathies are characterized by a progressive weakening of either proximal (MFM) or distal (DM) skeletal muscles combined with severe muscle defects [22, 23]. The disease spectrum has recently been broadened by the identification of novel genetic alterations in *FLNC* in patients with hypertrophic cardiomyopathy (HCM) [21]. Patients with HCM have marked sarcomeric abnormalities in cardiac muscles which cause an increased incidence of sudden cardiac death [51].

Based on the currently available data, we can suggest that the missense variants detected in *FLNC* are specific to the FTD phenotype based on a number of reasons. First, none of the *FLNC* variants identified in the Belgian FTD cohort were previously reported as a causal factor underlying any form of (cardio)myopathy. One exception is p.A2430V which was identified in one Belgian control individual, and had previously been reported in a patient with HCM [21]. In this latter study, however, the p.A2430V variation was classified as neutral by the SIFT prediciton software tool and considered a potential rare polymorphism in *FLNC,* which is supported by our observation of the p.A2430V variant in a Belgian control individual. Second, the *FLNC* carriers with FTD in our study did not show overt clinical signs of any form of myopathy. Considering that the average onset age of most FTD patients is older than of myopathy patients further strengthens our interpretation that the FLNC variants we observed are specific for FTD. Third, the missense variations in FTD are affecting evolutionary conserved regions across the actin binding domain (ABD) and the 24 Immunoglobulin (Ig)-like domains (Fig. 1b, Additional file 2: Figure S1). Although the pathogenicity of the *FLNC* variations remains speculative, p.V831I and p.R1241C showed alterations in the expression of the FLNC protein itself or its phosphorylation state. A number of FLNC variations - p.R81C, p.K524R, p.D710N, p.R1241C, and p.T2025I - were identified in FTD patients who carried a causal mutation in another FTD gene i.e. *FLNC* p.R1241C in a *VCP* p.R159H carrier, *FLNC* p.R81C and p.T2025I in *GRN* loss-of-function mutation carriers [10], and *FLNC* p.K524R and p.D710N in *C9orf72* repeat expansion carriers [11] (Table 2, Additional file 1: Table S3b). Co-occurrence of *FLNC* variants with causal *GRN* or *C9orf72* mutations is not surprising since they are the most frequent mutations found in FTD patients in Belgium. Also, *FLNC* p.E1571K was identified in a FTD patient carrying the *TREM2* p.R47H risk allele [52, 53]. Whether the presence of these FLNC missense variations may influence the pathological effect of causal mutations in *C9orf72* or *GRN*, or exert an additional pathological effect remains to be investigated. Overall, our genetic data obtained suggested that the role of *FLNC* variations might not be restricted to skeletal or cardiac muscle disorders, but might also be modifying the neurodegenerative processes in FTD patients. This implies that variations found in the same or in different functional domains of FLNC can be associated with distinct disease phenotypes involving miscellaneous tissues. This can be potentially explained by scaffolding functions of FLNC where different variants in certain protein domains could have differential effects on downstream signaling pathways or modulate distinct forms of FLNC protein-protein interactions.

Besides the moderate increase of FLNC levels in the frontal cortex of the *FLNC* p.V831I carrier, the *GRN* p.0(IVS1 + 5G > C) loss-of-function and to a lower extent the *VCP* p.R159H mutation showed a marked up-regulation of total FLNC levels. The potential link between GRN and FLNC was further supported by the strong increase in mouse Flnc levels observed in the frontal cortex of Grn^-/-^ mice between 16-18 months and 24 months of age. Hence it seems that in this FTD paradigm a strong aging-dependent triggering process may be in evidence.

Interestingly, the *VCP* p.R159H seemed to have a more pronounced effect on the phosphorylation state of pSer^2113^ while this was more the case for pSer^2213^ in GRN loss-of-function patients, suggesting that alternative pathways are affected in these patients. Increased phosphorylation at pSer^2113^ and pSer^2213^ of FLNC, might hamper the interaction of FLNC with other signaling proteins or its ability to modulate downstream signal transduction [49]. Further research, however, is required to determine the downstream functional discrepancies between elevated levels of total FLNC and both phosphorylation states of FLNC.

How elevated FLNC levels in the brain can influence the development of TDP-43 proteinopathies remains unknown. Studies performed in cellular and animal models, however, have demonstrated that a precise stoichiometry of FLNC together with its associated signaling/binding proteins is of critical importance for muscle function and maintenance [54]. Furthermore, it has been suggested that a certain threshold level of filamins may be essential for cell viability [14, 55]. In non-muscle cells, filamins are reported to colocalize along stress fibers implicating that they might play a role in maintaining focal adhesion complexes and stabilization of cytoskeletal features [56]. As no changes in both *Flna* and *Flnb* could be observed, the elevated levels of FLNC identified both in human and in mouse could hamper neuronal cellular integrity or axonal/dendritic plasticity in the brain to cope with additional stressors or alterations during degenerative and aging processes. This posit is in line with the Flnc expression patterns that we measured in the brains of Grn^-/-^mice. As mentioned previously, our expression data obtained from aged Grn^-/-^ mice demonstrated a strong increase of *Flnc* in brain between 16-18 months and 24 months. At an age of 23.5 months, the highest Flnc levels in mice also correlate with an 17.5-fold higher relative risk of dying for Grn^-/-^ mice compared to Grn^+/-^ mice [24]. This suggests that elevated Flnc levels might play an important role in mediating or accelerating the pathological aging process.

A potential role for accelerated aging and neurodegeneration, due to increased FLNC levels, is further supported by the identification of several protein factors in all differential datasets obtained from our exploratory quantitative proteomic analyses on the frontal cortex of a selected number of FTD patients with different genetic etiologies. For example, low levels of neurogranin show a clear correlation with cognitive deficits and aging [57]. The key role played by neuroplastins in synapse formation and its association with impaired intellectual ability might be indicative for the role of FLNC [58]. Furthermore, we identified also increased PGAM2 expression levels in the FLNC- and GRN-unique datasets as an additional muscle-specific protein that shows altered expression levels in the brain. Interestingly, PGAM2 overexpression has previously been associated with an altered energy metabolism and reduced stress resistance due to a decreased respiration capacity in mitochondria and increased production of reactive oxygen species in the heart of Pgam2 overexpression mice [59]. Abnormal energy metabolism has recently been linked to inflammation [60]. Furthermore, additional proteins which were consistently altered in both the FLNC-, GRN- and VCP-unique datasets, e.g. GFAP [61], NPTN [62], DBI [63], PRDX6 [64], MARCKS [65] and BSN [66], are closely associated with cognitive function, dementia and pathological aging, (Additional file 1: Table S6-8). Altered expression levels in these proteins could explain the observed accelerated aging of Grn^-/-^ mice and concomitant increase in Flnc, but clearly further research into this aging-neurodegenerative nexus in FTD is necessary.

Compared to the GRN- and VCP-unique datasets, the FLNC-unique protein dataset showed the strongest correlation to frontotemporal dementia, neurodegeneration and aging (Figs. 6 and 7). The bioinformatic analyses of all differential datasets provided evidence that the FLNC-unique protein dataset showed the greatest similarity to the core functions of the FTD-common protein dataset with respect to the modulation of neuronal structure or cytoskeletal dynamics (Figs. 5 and 6). Further support for the strong and specific connection between the core FTD functionality and the FLNC-specific dataset was provided by the application of standard bioinformatic investigations with GO Term and KEGG pathway analyses (Additional file 1: Table S17-20). The results of our exploratory proteomic analyses of frontal cortex of FTD patients are encouraging the involvement of FLNC in FTD disease pathways, however, further experiments are required to confirm these observations and should be extended on potential brain material of additional FTD patients with concomitant elevated FLNC levels.

Hence we found four common significantly populated GO terms between the FTD-common and FLNC-specific dataset, i.e. *oxidative phosphorylation, generation of precursor metabolites and energy, cellular protein complex assembly* and *protein polymerization*. Only one common GO term could be found between the GRN-unique (*transmembrane transport*) or VCP-unique (*ribonucleotide metabolic process*) datasets and the FTD-common dataset. At the KEGG pathway level, four common pathways were identified between the FTD-common and the FLNC-unique dataset: *oxidative phosphorylation, Parkinson's disease, Alzheimer's disease, pathogenic Escherichia coli infection*. No common pathways were found between the GRN- or VCP-unique datasets and the FTD-common dataset. In both cases the bioinformatic interpretation of the FLNC-unique dataset reveals a strong neurometabolic, neuronal architectural and neuordegenerative functional bias.

## Conclusions

The data presented here provide further support that FLNC, a muscle-specific protein, could be a potential novel player in FTD pathogenesis. More specifically, we report a significant association between rare variants in *FLNC* and FTD. We have demonstrated that elevated FLNC levels in the frontal cortex of FTD patients are mainly associated with GRN haploinsufficiency and to a lower extent to the *FLNC* p.V831I and *VCP* p.R159H mutation. The *GRN* associated increase of FLNC was confirmed in the frontal cortex of aged Grn knockout mice. Moreover, proteomic analysis of FTD patients with increased FLNC levels points towards downstream alterations in pathways involved in aging, neurodegeneration and synaptogenesis, suggesting that FLNC levels might have a potential role in mediating or accelerating the aging process. However, the exact mode of action of increased FLNC levels in the brain of FTD patients is currently unknown and requires further investigation.

## Additional files

Additional file 1: Supplementary tables. (DOC 1000 kb) Additional file 2: Supplementary figures. (DOC 2024 kb)
