# Supplementary material for: Investigating the role of filamin C in Belgian patients with frontotemporal dementia linked to GRN deficiency in FTLD-TDP brains
Source: Acta Neuropathol Commun. 2015 Nov 10;3:68. doi: 10.1186/s40478-015-0246-7 (PMC4641381; doi:10.1186/s40478-015-0246-7)
Supplement: Additional file 1: Supplementary tables. — (DOC 1000 kb) [file 40478_2015_246_MOESM1_ESM.doc]

### **Supplementary Tables**

| **Supplementary Table 1.** Antibodies used in FLNC expression analyses | | | | | |
| --- | --- | --- | --- | --- | --- |
| **Antibody** | **Epitope/marker** | **Species** | **Clonality** | **Dilution** | **Source** |
| anti-human FLNC | Filamin C | Rabbit | pAb | 1/5000 | Kunkel lab |
| anti-mouse FLNC | Filamin C | Rabbit | pAb | 1/500 | Kinasource |
| anti-pSer^2113^ FLNC | Filamin C | Rabbit | pAb | 1/1000 | Life Technologies |
| anti-pSer^2213^ FLNC | Filamin C | Sheep | pAb | 1/1000 | Kinasource |
| anti-GAPDH | Glyceraldehyde 3-phosphate dehydrogenase | Rabbit | pAb | 1/20000 | GeneTex |
| anti-PCDGF | PC cell-derived growth factor, progranulin | Rabbit | pAb | 1/1000 | Life Technologies |
| anti-PGAM2 | Phosphoglycerate mutase 2 | Rabbit | mAb | 1/1000 | Abcam |
| Anti-STXBP2 | Syntaxin-binding protein 2 | Rabbit | pAb | 1/1000 | GeneTex |

| **Supplementary Table 2.** Overview of GRN siRNAs and control siRNAs used in knockdown experiments | | | | |
| --- | --- | --- | --- | --- |
| **Label** | **Target** | **siRNA sequence (5' – 3')** | **Company (ID)** | **Concentration** |
| siRNA GRN_1 | Progranulin | GGUGCUUUUCCCUAUCCACtt | Ambion (ID 144870) | 20 nM |
| siRNA GRN_2 | Progranulin | CGUGCUGUGUUAUGGUCGAtt | Ambion (ID 144869) | 20 nM |
| siRNA GRN_3 | Progranulin | GGGACAGUACUGAAGACUCtt | Ambion (ID 11032) | 20 nM |
| siRNA Scrambled | Non-targeting | not provided | Qiagen (N°1027281) | 20 nM |

| **Supplementary Table 3a:** Rare FLNC variants (MAF <1%) identified in patient and control individuals. | | | | |
| --- | --- | --- | --- | --- |
| **Variant** | **Functional domain** | **FTD** | **Controls** | **dbSNP** |
| p.M370V | Ig 2 | 1 | 1 | rs370406338 |
| p.K492E | Ig 3 | 2 | 1 | rs118056738 |
| p.G507R | Ig 3 | 4 | 3 | rs189525930 |
| p.R526Q | Ig 3 | 3 | 2 | rs34932223 |
| p.D693A | Ig 5 | 7 | 3 | rs34972246 |
| p.T834M | Ig 6 | 2 | 3 | rs75133741 |
| p.W1766R | Ig 16 | 1 | 1 | - |
| p.P1771A | Ig 16 | 2 | 1 | rs200001272 |
| p.R1860C | Ig 17 | 8 | 11 | rs181067717 |
| p.I1882V | Ig 17 | 4 | 7 | rs184018403 |
| p.T1943I | Ig 17 | 1 | 1 | rs376413798 |
| p.V2059M | Ig 19 | 1 | 3 | rs201333104 |
| p.E2270K | Ig 20 | 3 | 2 | rs202223616 |
|  |  |  |  |  |
| **Supplementary Table 3b:** FLNC variants (MAF >1%) identified in patient and control individuals. | | | | |
| **Variant** | **Functional domain** | **FTD** | **Controls** | **dbSNP** |
| p.R1241C | Ig 10 | 9 | 24 | rs146953558 |
| p.R1567Q | Ig 14 | 52 | 136 | rs2291569 |

| **Supplementary Table 4:** Rare FLNC variants (MAF <1%) identified in control individuals only. | | |
| --- | --- | --- |
| **Variant** | **Functional domain** | **dbSNP** |
| p.R361C | Ig 1 | rs200206944 |
| p.N393K | Ig 2 | - |
| p.A443V | Ig 2 | - |
| p.C472R | Ig 3 | - |
| p.V523A | Ig 3 | rs182845462 |
| p.E534K | Ig 3 | rs201905890 |
| p.F690L | Ig 5 | rs200943714 |
| p.A709T | Ig 5 | rs192725607 |
| p.G722S | Ig 5 | - |
| p.R755W | Ig 5 | - |
| p.G876V | Ig 7 | - |
| p.G885R | Ig 7 | - |
| p.V892A | Ig 7 | - |
| p.F1153C | Ig 10 | rs138663492 |
| p.P1155L | Ig 10 | - |
| p.R1167C | Ig 10 | - |
| p.A1208V | Ig 10 | rs202184162 |
| p.S1251G | Ig 11 | - |
| p.S1296L | Ig 11 | - |
| p.V1314G | Ig 11 | - |
| p.R1434C | Ig 12 | - |
| p.A1475V | Ig 13 | rs369305865 |
| p.R1526C | Ig 13 | - |
| p.A1588G | Ig 14 | rs148545460 |
| p.A1792T | Ig 16 | rs201348102 |
| p.R1931C | Ig 17 | - |
| p.R1982C | Ig 18 | - |
| p.S1985L | Ig 18 | rs200415625 |
| p.R2230H | linker Ig 19-20 | rs376035195 |
| p.A2273T | Ig 20 | rs372251350 |
| p.R2364H | Ig 21 | rs201672146 |
| p.A2430V | Ig 22 | rs200516164 |
| p.V2431M | Ig 22 | - |
| p.D2551G | Ig 23 | - |

| **Supplementary Table 5a**: In silico predictions of rare FLNC missense variants (MAF < 1%) identified in the Belgian FTLD cohort. | | | | | | |
| --- | --- | --- | --- | --- | --- | --- |
| **Variant** | **SIFT** | | **PolyPhen-2** | | **SNAP** | |
|  | **Prediction** | **SIFT Score** | **Prediction** | **PolyPhen-2 Score** | **Prediction** | **Expected Accuracy** |
| p.R81C | Damaging | 0 | Possibly Damaging | 0.954 | Non-neutral | 71% |
| p.E309K | Damaging | 0.05 | Benign | 0.381 | Non-neutral | 66% |
| p.E323V | Damaging | 0 | Probably Damaging | 0.992 | Non-neutral | 63% |
| p.K524R | Damaging | 0 | Probably Damaging | 0.996 | Neutral | 87% |
| p.D710N | Tolerated | 0.07 | Possibly Damaging | 0.538 | Neutral | 57% |
| p.A806T | Tolerated | 0.8 | Possibly Damaging | 0.538 | Neutral | 93% |
| p.V831I | Damaging | 0.04 | Probably Damaging | 1,000 | Non-neutral | 59% |
| p.V1047L | Damaging | 0 | Possibly Damaging | 0.932 | Non-neutral | 75% |
| p.P1163R | Damaging | 0 | Probably Damaging | 1,000 | Non-neutral | 75% |
| p.V1335M | Damaging | 0 | Possibly Damaging | 0.832 | Non-neutral | 71% |
| p.L1364F | Tolerated | 0.72 | Benign | 0.019 | Neutral | 82% |
| p.R1370Q | Tolerated | 0.57 | Benign | 0.002 | Neutral | 57% |
| p.A1551T | Tolerated | 1 | Benign | 0.001 | Neutral | 97% |
| p.E1571K | Tolerated | 0.19 | Benign | 0.023 | Neutral | 87% |
| p.R1758W | Damaging | 0.01 | Benign | 0,000 | Non-neutral | 59% |
| p.V2014A | Tolerated | 0.16 | Benign | 0.35 | Neutral | 97% |
| p.T2025I | Damaging | 0 | Possibly Damaging | 0.566 | Neutral | 71% |
| p.R2318Q | Tolerated | 0.37 | Probably Damaging | 0.999 | Neutral | 61% |
| p.S2461N | Tolerated | 0.28 | Benign | 0.001 | Neutral | 87% |

*SIFT predicts substitutions with scores less than 0.05 as deleterious. The PolyPhen-2 score indicates the probability of a deleterious effect (1 has*

*high confidence).*

| **Supplementary Table 5b**: In silico predictions of rare FLNC missense variants (MAF < 1%) identified in patient and control individuals. | | | | | | |
| --- | --- | --- | --- | --- | --- | --- |
| **Variant** | **SIFT** | | **PolyPhen-2** | | **SNAP** | |
|  | **Prediction** | **SIFT Score** | **Prediction** | **PolyPhen-2 Score** | **Prediction** | **Expected Accuracy** |
| p.M370V | Tolerated | 0.18 | Benign | 0.033 | Neutral | 53% |
| p.K492E | Damaging | 0.02 | Possibly Damaging | 0.537 | Non-neutral | 66% |
| p.G507R | Damaging | 0 | Probably Damaging | 1.000 | Non-neutral | 80% |
| p.R526Q | Tolerated | 0.08 | Benign | 0.027 | Neutral | 78% |
| p.D693A | Damaging | 0 | Possibly Damaging | 0.923 | Non-neutral | 66% |
| p.T834M | Tolerated | 0.15 | Benign | 0.249 | Neutral | 88% |
| p.W1766R | Tolerated | 0.32 | Benign | 0.009 | Non-neutral | 75% |
| p.P1771A | Tolerated | 0.6 | Benign | 0.000 | Neutral | 78% |
| p.R1860C | Damaging | 0.01 | Probably Damaging | 0.961 | Non-neutral | 59% |
| p.I1882V | Tolerated | 0.75 | Benign | 0.010 | Neutral | 88% |
| p.T1943I | Damaging | 0.01 | Benign | 0.392 | Non-neutral | 63% |
| p.V2059M | Tolerated | 0.13 | Benign | 0.044 | Neutral | 87% |
| p.E2270K | Tolerated | 0.73 | Benign | 0.371 | Neutral | 72% |

*SIFT predicts substitutions with scores less than 0.05 as deleterious. The PolyPhen-2 score indicates the probability of a deleterious*

*effect (1 has high confidence).*

| **Supplementary Table 5c**: In silico predictions of rare FLNC missense variants (MAF < 1%) identified in the Belgian control cohort. | | | | | | |
| --- | --- | --- | --- | --- | --- | --- |
| **Variant** | **SIFT** | | **PolyPhen-2** | | **SNAP** | |
|  | **Prediction** | **SIFT Score** | **Prediction** | **PolyPhen-2 Score** | **Prediction** | **Expected Accuracy** |
| p.R361C | Damaging | 0 | Probably damaging | 0.977 | Non-neutral | 75% |
| p.C472R | Damaging | 0.01 | Possibly damaging | 0.737 | Non-neutral | 80% |
| p.N393K | Tolerated | 0.11 | Possibly damaging | 0.841 | Neutral | 53% |
| p.A443V | Tolerated | 0.72 | Benign | 0.000 | Neutral | 93% |
| p.V523A | Damaging | 0.03 | Possibly damaging | 0.680 | Neutral | 66% |
| p.E534K | Tolerated | 0.65 | Benign | 0.028 | Neutral | 78% |
| p.F690L | Damaging | 0 | Probably damaging | 1.000 | Non-neutral | 71% |
| p.A709T | Tolerated | 0.07 | Possibly damaging | 0.953 | Neutral | 53% |
| p.G722S | Tolerated | 0.13 | Probably damaging | 0.987 | Neutral | 61% |
| p.R755W | Damaging | 0 | Probably damaging | 1.000 | Non-neutral | 80% |
| p.G876V | Damaging | 0 | Probably damaging | 1.000 | Non-neutral | 71% |
| p.G885R | Damaging | 0.02 | Possibly damaging | 0.938 | Non-neutral | 75% |
| p.V892A | Damaging | 0 | Probably damaging | 0.973 | Non-neutral | 71% |
| p.F1153C | Tolerated | 0.2 | Probably damaging | 1.000 | Neutral | 61% |
| p.P1155L | Damaging | 0 | Probably damaging | 0.974 | Non-neutral | 80% |
| p.R1167C | Damaging | 0.01 | Benign | 0.033 | Non-neutral | 71% |
| p.A1208V | Tolerated | 0.09 | Possibly damaging | 0.536 | Neutral | 72% |
| p.S1251G | Damaging | 0.04 | Probably damaging | 0.985 | Neutral | 66% |
| p.S1296L | Damaging | 0 | Probably damaging | 1.000 | Neutral | 66% |
| p.V1314G | Damaging | 0 | Probably damaging | 0.977 | Non-neutral | 80% |
| p.R1434C | Damaging | 0 | Probably damaging | 0.999 | Non-neutral | 75% |
| p.A1475V | Damaging | 0 | Probably damaging | 1.000 | Non-neutral | 53% |
| p.R1526C | Damaging | 0.04 | Benign | 0.208 | Neutral | 53% |
| p.A1588G | Damaging | 0 | Possibly damaging | 0.863 | Neutral | 66% |
| p.A1792T | Tolerated | 1 | Benign | 0.002 | Neutral | 87% |
| p.R1931C | Damaging | 0 | Probably damaging | 0.999 | Non-neutral | 66% |
| p.R1982C | Damaging | 0.04 | Probably damaging | 0.983 | Non-neutral | 71% |
| p.S1985L | Damaging | 0 | Probably damaging | 1.000 | Neutral | 53% |
| p.R2230H | Damaging | 0.01 | Possibly damaging | 0.832 | Neutral | 57% |
| p.A2273T | Tolerated | 0.27 | Benign | 0.006 | Neutral | 97% |
| p.R2364H | Damaging | 0.03 | Benign | 0.061 | Neutral | 61% |
| p.A2430V | Tolerated | 0.41 | Possibly damaging | 0.871 | Neutral | 93% |
| p.V2431M | Damaging | 0 | Probably damaging | 0.998 | Non-neutral | 75% |
| p.D2551G | Tolerated | 0.08 | Probably damaging | 0.999 | Neutral | 61% |

*SIFT predicts substitutions with scores less than 0.05 as deleterious. The PolyPhen-2 score indicates the probability of a deleterious effect (1 has high confidence).*

**Supplementary Table 6. Cortical proteins significantly altered in expression between *FLNC* p.V831I and multiple control individuals.** Primary iTRAQ expression ratios were Log_2_ transformed. Proteins represented in the table demonstrated a significant divergence in expression level (*P* <0.05) compared to the global mean protein expression level. For each significantly-regulated protein the associated Uniprot protein accession number, protein description and log_2_-transformed expression ratio are displayed.

| **Protein Accession** | **Protein Description** | **Log_2_ iTRAQ Ratio** |
| --- | --- | --- |
| P15259 | phosphoglycerate mutase 2 | 1.922712155 |
| O95372 | acyl-protein thioesterase 2 | 1.333423734 |
| P08123 | Collagen alpha-2(I) chain | 1.23878686 |
| A8MUU1 | Putative fatty acid-binding protein 5-like protein 3 | 1.15704371 |
| Q86UD0 | Suppressor APC domain-containing protein 2 | 1.124267418 |
| P02452 | Collagen alpha-1(I) chain | 1.084048215 |
| Q01469 | Fatty acid-binding protein, epidermal | 1.042644337 |
| P14136 | Glial fibrillary acidic protein | 1.007195501 |
| P14136-2 | Isoform 2 of Glial fibrillary acidic protein | 0.992768431 |
| P30041 | Peroxiredoxin-6 | 0.941106311 |
| Q8N8S7 | Protein enabled homolog | 0.933572638 |
| Q8N8S7-2 | Isoform 2 of Protein enabled homolog | 0.933572638 |
| Q8N8S7-3 | Isoform 3 of Protein enabled homolog | 0.933572638 |
| P07951-1 | Tropomyosin beta chain | 0.895302621 |
| O00483 | Cytochrome c oxidase subunit NDUFA4 | 0.871843649 |
| O43298 | Zinc finger and BTB domain-containing protein 43 | 0.839959587 |
| O75121 | Microfibrillar-associated protein 3-like | 0.831877241 |
| O75121-2 | Isoform 2 of Microfibrillar-associated protein 3-like | 0.831877241 |
| P55082-1 | Microfibril-associated glycoprotein 3 | 0.831877241 |
| P98160 | Basement membrane-specific heparan sulfate proteoglycan core protein | 0.831877241 |
| Q2LD37-1 | Uncharacterized protein KIAA1109 | 0.82374936 |
| Q9UII2-2 | Isoform 2 of ATPase inhibitor, mitochondrial | 0.82374936 |
| Q9UII2-3 | Isoform 3 of ATPase inhibitor, mitochondrial | 0.82374936 |
| Q9UPA5 | Protein bassoon | 0.82374936 |
| Q9UPY6 | Wiskott-Aldrich syndrome protein family member 3 | 0.82374936 |
| P07108 | acyl-CoA-binding protein | 0.815575429 |
| P22570 | NADPH:adrenodoxin oxidoreductase, mitochondrial | 0.815575429 |
| P09488 | Glutathione S-transferase Mu 1 | 0.807354922 |
| Q03013-1 | glutathione S-transferase mu 4 | 0.807354922 |
| Q14558 | Phosphoribosyl pyrophosphate synthase-associated protein 1 | 0.807354922 |
| Q14558-2 | Isoform 2 of Phosphoribosyl pyrophosphate synthase-associated protein 1 | 0.807354922 |
| Q13162 | Peroxiredoxin-4 | 0.799087306 |
| P31321 | cAMP-dependent protein kinase type I-beta regulatory subunit | 0.790772038 |
| Q9UII2-1 | ATPase inhibitor, mitochondrial | 0.782408565 |
| P02792 | Ferritin light chain | 0.773996325 |
| Q9HC56 | Protocadherin-9 | 0.765534746 |
| O00625 | Pirin | 0.748461233 |
| P05230-1 | Fibroblast growth factor 1 | 0.748461233 |
| P05230-2 | Isoform 2 of Fibroblast growth factor 1 | 0.748461233 |
| P20774 | Mimecan | 0.748461233 |
| Q14019 | coactosin-like protein | 0.748461233 |
| Q5H9L2 | Transcription elongation factor A protein-like 5 | 0.748461233 |
| Q5TC84 | Opioid growth factor receptor-like protein 1 | 0.748461233 |
| Q969E4 | transcription elongation factor A protein-like 3 | 0.748461233 |
| Q9BQ24 | Zinc finger FYVE domain-containing protein 21 | 0.748461233 |
| Q9BQ24-2 | Isoform 2 of Zinc finger FYVE domain-containing protein 21 | 0.748461233 |
| P23142 | Fibulin-1 | 0.731183242 |
| P30622-3 | CAP-Gly domain-containing linker protein 1 | 0.731183242 |
| P17302 | Gap junction alpha-1 protein | 0.722466024 |
| P50452 | Serpin B8 | 0.722466024 |
| P50452-2 | Isoform 2 of Serpin B8 | 0.722466024 |
| P03915 | NADH-ubiquinone oxidoreductase chain 5 | 0.713695815 |
| P50135 | Histamine N-methyltransferase | 0.713695815 |
| Q6NVY1 | 3-hydroxyisobutyryl-CoA hydrolase, mitochondrial | 0.713695815 |
| Q6NVY1-2 | Isoform 2 of 3-hydroxyisobutyryl-CoA hydrolase, mitochondrial | 0.713695815 |
| Q9NZ56 | Formin-2 | 0.713695815 |
| P29972-1 | aquaporin-1 | 0.704871964 |
| P46940 | Ras GTPase-activating-like protein IQGAP1 | 0.704871964 |
| Q06323-1 | Proteasome activator complex subunit 1 | 0.695993813 |
| Q9GZV7 | Hyaluronan and proteoglycan link protein 2 | 0.695993813 |
| Q6P1X6 | UPF0598 protein C8orf82 | 0.687060688 |
| Q92820 | Gamma-glutamyl hydrolase | 0.687060688 |
| Q9P2R3 | Rabankyrin-5 | 0.687060688 |
| P14136-3 | Isoform 3 of Glial fibrillary acidic protein | 0.678071905 |
| P20472 | Parvalbumin alpha | 0.678071905 |
| P29966 | Myristoylated alanine-rich C-kinase substrate | 0.678071905 |
| Q8N7J2-1 | APC membrane recruitment protein 2 | 0.678071905 |
| P04271 | Protein S100-B | 0.669026766 |
| O14745 | Na(+)/H(+) exchange regulatory cofactor NHE-RF1 | 0.659924558 |
| O43581-2 | Isoform 2 of Synaptotagmin-7 | 0.659924558 |
| P07203-2 | Isoform 2 of Glutathione peroxidase 1 | 0.659924558 |
| P69891 | Hemoglobin subunit gamma-1 | 0.659924558 |
| P69892 | Hemoglobin subunit gamma-2 | 0.659924558 |
| Q2TB90-1 | Putative hexokinase HKDC1 | 0.659924558 |
| Q58FG1 | Putative heat shock protein HSP 90-alpha A4 | 0.659924558 |
| Q8N196 | homeobox protein SIX5 | 0.659924558 |
| Q9BX66-1 | Sorbin and SH3 domain-containing protein 1 | 0.659924558 |
| Q9NQW7-1 | xaa-Pro aminopeptidase 1 | 0.659924558 |
| B2RPK0 | Putative high mobility group protein B1-like 1 | 0.650764559 |
| P09429 | High mobility group protein B1 | 0.650764559 |
| P23497 | Nuclear autoantigen Sp-100 | 0.650764559 |
| Q8WZA0 | Protein LZIC | 0.650764559 |
| Q9Y617-1 | phosphoserine aminotransferase | 0.650764559 |
| Q9Y617-2 | Isoform 2 of Phosphoserine aminotransferase | 0.650764559 |
| O95299 | NADH dehydrogenase [ubiquinone] 1 alpha subcomplex subunit 10, mitochondrial | 0.641546029 |
| O95299-2 | Isoform 2 of NADH dehydrogenase [ubiquinone] 1 alpha subcomplex subunit 10, mitochondrial | 0.641546029 |
| P13611-2 | Isoform V1 of Versican core protein | 0.641546029 |
| P13611-4 | Isoform V3 of Versican core protein | 0.641546029 |
| P42330 | Aldo-keto reductase family 1 member C3 | 0.641546029 |
| P42330-2 | Isoform 2 of Aldo-keto reductase family 1 member C3 | 0.641546029 |
| P57058 | Hormonally up-regulated neu tumor-associated kinase | 0.641546029 |
| Q06830 | peroxiredoxin-1 | 0.641546029 |
| Q8NFZ8 | Cell adhesion molecule 4 | 0.641546029 |
| Q9UIJ7-2 | Isoform 2 of GTP:AMP phosphotransferase AK3, mitochondrial | 0.641546029 |
| P13611 | Versican core protein | 0.632268215 |
| P13611-3 | Isoform V2 of Versican core protein | 0.632268215 |
| P13611-5 | Isoform Vint of Versican core protein | 0.632268215 |
| P02533 | Keratin, type I cytoskeletal 14 | 0.613531653 |
| P08779 | Keratin, type I cytoskeletal 16 | 0.613531653 |
| Q7Z3Y9 | Keratin, type I cytoskeletal 26 | 0.613531653 |
| O00499-2 | Isoform IIB of Myc box-dependent-interacting protein 1 | 0.584962501 |
| Q6KB66 | Keratin, type II cytoskeletal 80 | 0.584962501 |
| P31689-1 | DnaJ homolog subfamily A member 1 | 0.575312331 |
| P31689-2 | Isoform 2 of DnaJ homolog subfamily A member 1 | 0.575312331 |
| Q9BW30 | Tubulin polymerization-promoting protein family member 3 | 0.575312331 |
| P20916-1 | Myelin-associated glycoprotein | 0.565597176 |
| P21810 | biglycan | 0.555816155 |
| Q9Y696 | Chloride intracellular channel protein 4 | 0.545968369 |
| O60234 | glia maturation factor gamma | 0.526068812 |
| Q3SY69-1 | Mitochondrial 10-formyltetrahydrofolate dehydrogenase | 0.526068812 |
| P19013 | Keratin, type II cytoskeletal 4 | 0.516015147 |
| P21266 | glutathione S-transferase Mu 3 | 0.495695163 |
| P60201-2 | Isoform DM-20 of Myelin proteolipid protein | 0.495695163 |
| P06703 | protein S100-A6 | 0.485426827 |
| P51888 | prolargin | 0.485426827 |
| P61328-1 | Fibroblast growth factor 12 | 0.485426827 |
| Q14CZ8-2 | Isoform 2 of Hepatocyte cell adhesion molecule | 0.485426827 |
| Q92915 | fibroblast growth factor 14 | 0.485426827 |
| Q99584 | Protein S100-A13 | 0.485426827 |
| Q9H1E3 | Nuclear ubiquitous casein and cyclin-dependent kinase substrate 1 | 0.485426827 |
| Q9H1E3-2 | Isoform 2 of Nuclear ubiquitous casein and cyclin-dependent kinase substrate 1 | 0.485426827 |
| Q9Y2J8 | Protein-arginine deiminase type-2 | 0.485426827 |
| P35749 | Myosin-11 | 0.475084883 |
| P49189 | 4-trimethylaminobutyraldehyde dehydrogenase | 0.475084883 |
| Q9UHC1-1 | DNA mismatch repair protein MLH3 | 0.464668267 |
| Q9UHC1-2 | Isoform 2 of DNA mismatch repair protein Mlh3 | 0.464668267 |
| Q9ULH1-1 | Arf-GAP with SH3 domain, ANK repeat and PH domain-containing protein 1 | 0.464668267 |
| P02008 | Hemoglobin subunit zeta | 0.454175893 |
| P49189-2 | Isoform 2 of 4-trimethylaminobutyraldehyde dehydrogenase | 0.454175893 |
| Q8TCD5-2 | Isoform 2 of 5'(3')-deoxyribonucleotidase, cytosolic type | 0.454175893 |
| Q8N6N7 | acyl-CoA-binding domain-containing protein 7 | 0.443606651 |
| Q9NZ01-1 | Very-long-chain enoyl-CoA reductase | 0.432959407 |
| O43175 | D-3-phosphoglycerate dehydrogenase | 0.422233001 |
| P07741-2 | Isoform 2 of Adenine phosphoribosyltransferase | 0.422233001 |
| P08758 | annexin A5 | 0.422233001 |
| P09382 | Galectin-1 | 0.422233001 |
| Q13938-3 | Isoform 2 of Calcyphosin | 0.411426246 |
| Q9H0N5 | Pterin-4-alpha-carbinolamine dehydratase 2 | 0.411426246 |
| Q9ULV4 | coronin-1C | 0.411426246 |
| P00739-1 | Haptoglobin-related protein | 0.40053793 |
| P49419-3 | Isoform 3 of Alpha-aminoadipic semialdehyde dehydrogenase | 0.40053793 |
| Q9Y2S2-1 | Lambda-crystallin homolog | 0.40053793 |
| P07197-2 | Isoform 2 of Neurofilament medium polypeptide | -0.01449957 |
| Q7L1I2-2 | Isoform 2 of Synaptic vesicle glycoprotein 2B | -0.01449957 |
| Q9BRF8-1 | Serine/threonine-protein phosphatase CPPED1 | -0.01449957 |
| O15400 | Syntaxin-7 | -0.029146346 |
| Q15819 | Ubiquitin-conjugating enzyme E2 variant 2 | -0.029146346 |
| Q8NC96 | adaptin ear-binding coat-associated protein 1 | -0.029146346 |
| Q13748-1 | tubulin alpha-3C/D chain | -0.043943348 |
| Q13748-2 | Isoform 2 of Tubulin alpha-3C/D chain | -0.043943348 |
| Q6PEY2 | Tubulin alpha-3E chain | -0.043943348 |
| P07197 | Neurofilament medium polypeptide | -0.058893689 |
| P09936 | Ubiquitin carboxyl-terminal hydrolase isozyme L1 | -0.058893689 |
| P27816-7 | Isoform 7 of Microtubule-associated protein 4 | -0.058893689 |
| Q71U36 | tubulin alpha-1A chain | -0.058893689 |
| Q9BQE3 | Tubulin alpha-1C chain | -0.058893689 |
| Q9UD71-1 | Protein phosphatase 1 regulatory subunit 1B | -0.058893689 |
| Q15067 | Peroxisomal acyl-coenzyme A oxidase 1 | -0.074000581 |
| Q5K651 | Sterile alpha motif domain-containing protein 9 | -0.074000581 |
| Q8IVG5 | sterile alpha motif domain-containing protein 9-like | -0.074000581 |
| P26640-2 | Isoform 2 of Valine--tRNA ligase | -0.089267338 |
| Q9BWS9-1 | Chitinase domain-containing protein 1 | -0.089267338 |
| P68363 | Tubulin alpha-1B chain | -0.104697379 |
| P30419 | glycylpeptide N-tetradecanoyltransferase 1 | -0.120294234 |
| P30419-2 | Isoform Short of Glycylpeptide N-tetradecanoyltransferase 1 | -0.120294234 |
| P01034 | Cystatin-C | -0.152003093 |
| Q8TDB8-3 | Isoform 3 of Solute carrier family 2, facilitated glucose transporter member 14 | -0.152003093 |
| P07196 | Neurofilament light polypeptide | -0.168122759 |
| O43181 | NADH dehydrogenase [ubiquinone] iron-sulfur protein 4, mitochondrial | -0.268816758 |
| P49821 | NADH dehydrogenase [ubiquinone] flavoprotein 1, mitochondrial | -0.268816758 |
| Q08211-2 | Isoform 2 of ATP-dependent RNA helicase A | -0.268816758 |
| Q13596 | Sorting nexin-1 | -0.268816758 |
| Q13596-2 | Isoform 1A of Sorting nexin-1 | -0.268816758 |
| Q13596-3 | Isoform 3 of Sorting nexin-1 | -0.268816758 |
| Q7Z6L0-1 | Proline-rich transmembrane protein 2 | -0.268816758 |
| Q9NZ45 | CDGSH iron-sulfur domain-containing protein 1 | -0.268816758 |
| Q9UQM7-1 | Calcium/calmodulin-dependent protein kinase type II subunit alpha | -0.268816758 |
| Q86UW8 | Hyaluronan and proteoglycan link protein 4 | -0.286304185 |
| Q9BWQ8 | protein lifeguard 2 | -0.286304185 |
| Q9NUB1 | Acetyl-coenzyme A synthetase 2-like, mitochondrial | -0.286304185 |
| O00629 | Importin subunit alpha-3 | -0.304006187 |
| O43583 | density-regulated protein | -0.304006187 |
| P01877 | Ig alpha-2 chain C region | -0.304006187 |
| P04216 | Thy-1 membrane glycoprotein | -0.304006187 |
| P63215 | Guanine nucleotide-binding protein G(I)/G(S)/G(O) subunit gamma-3 | -0.304006187 |
| P68371 | Tubulin beta-4B chain | -0.304006187 |
| Q15067-3 | Isoform 3 of Peroxisomal acyl-coenzyme A oxidase 1 | -0.304006187 |
| Q7L1I2 | Synaptic vesicle glycoprotein 2B | -0.304006187 |
| Q8N196 | homeobox protein SIX5 | -0.304006187 |
| Q9NUB1 | Acetyl-coenzyme A synthetase 2-like, mitochondrial | -0.304006187 |
| Q9NZQ3-1 | NCK-interacting protein with SH3 domain | -0.304006187 |
| Q9UHD1 | cysteine and histidine-rich domain-containing protein 1 | -0.304006187 |
| Q9UHD1-2 | Isoform 2 of Cysteine and histidine-rich domain-containing protein 1 | -0.304006187 |
| Q9Y570 | Protein phosphatase methylesterase 1 | -0.304006187 |
| O95670-2 | Isoform 2 of V-type proton ATPase subunit G 2 | -0.321928095 |
| P07437 | tubulin beta chain | -0.321928095 |
| P61981 | 14-3-3 protein gamma | -0.321928095 |
| Q16718 | NADH dehydrogenase [ubiquinone] 1 alpha subcomplex subunit 5 | -0.321928095 |
| Q8NBX0 | saccharopine dehydrogenase-like oxidoreductase | -0.321928095 |
| Q99719-1 | Septin-5 | -0.321928095 |
| Q9Y487 | V-type proton ATPase 116 kDa subunit a isoform 2 | -0.321928095 |
| P01860 | Ig gamma-3 chain C region | -0.340075442 |
| P55263-1 | Adenosine kinase | -0.340075442 |
| P55263-2 | Isoform 2 of Adenosine kinase | -0.340075442 |
| P55263-4 | Isoform 4 of Adenosine kinase | -0.340075442 |
| P61966 | AP-1 complex subunit sigma-1A | -0.340075442 |
| P61966-2 | Isoform 2 of AP-1 complex subunit sigma-1A | -0.340075442 |
| Q9H1N7-1 | Adenosine 3'-phospho 5'-phosphosulfate transporter 2 | -0.340075442 |
| Q9H1N7-3 | Isoform 3 of Adenosine 3'-phospho 5'-phosphosulfate transporter 2 | -0.340075442 |
| Q9Y639-2 | Neuroplastin | -0.340075442 |
| P01861 | Ig gamma-4 chain C region | -0.358453971 |
| P05496 | ATP synthase F(0) complex subunit C1, mitochondrial | -0.358453971 |
| P08247 | Synaptophysin | -0.358453971 |
| P48201 | ATP synthase F(0) complex subunit C3, mitochondrial | -0.358453971 |
| P56378-1 | 6.8 kDa mitochondrial proteolipid | -0.358453971 |
| Q06055 | ATP synthase F(0) complex subunit C2, mitochondrial | -0.358453971 |
| Q9Y639-1 | Isoform 1 of Neuroplastin | -0.358453971 |
| Q9Y639-3 | Isoform 3 of Neuroplastin | -0.358453971 |
| P04156-1 | Major prion protein | -0.377069649 |
| P04156-2 | Isoform 2 of Major prion protein | -0.377069649 |
| P04350 | Tubulin beta-4A chain | -0.377069649 |
| P05155 | Plasma protease C1 inhibitor | -0.377069649 |
| P68366 | Tubulin alpha-4A chain | -0.377069649 |
| P68366-2 | Isoform 2 of Tubulin alpha-4A chain | -0.377069649 |
| Q15818 | Neuronal pentraxin-1 | -0.377069649 |
| P08247-2 | Isoform 2 of Synaptophysin | -0.395928676 |
| P16104 | Histone H2AX | -0.395928676 |
| Q8IUE6 | Histone H2A type 2-B | -0.395928676 |
| Q96QV6 | Histone H2A type 1-A | -0.395928676 |
| P13591-6 | Isoform 6 of Neural cell adhesion molecule 1 | -0.415037499 |
| P41732 | Tetraspanin-7 | -0.415037499 |
| Q6NXT2 | Histone H3.3C | -0.415037499 |
| Q99726 | Zinc transporter 3 | -0.415037499 |
| Q9UD71-2 | Isoform 2 of Protein phosphatase 1 regulatory subunit 1B | -0.415037499 |
| P11234 | Ras-related protein Ral-B | -0.434402824 |
| P49802-1 | regulator of G-protein signaling 7 | -0.434402824 |
| P80723 | Brain acid soluble protein 1 | -0.434402824 |
| A6NNZ2 | Tubulin beta-8 chain-like protein LOC260334 | -0.454031631 |
| P62249 | 40S ribosomal protein S16 | -0.454031631 |
| P62328 | Thymosin beta-4 | -0.454031631 |
| P63313 | thymosin beta-10 | -0.454031631 |
| Q15904 | V-type proton ATPase subunit S1 | -0.454031631 |
| Q3ZCM7 | tubulin beta-8 chain | -0.454031631 |
| P08754 | Guanine nucleotide-binding protein G(k) subunit alpha | -0.473931188 |
| P27449 | V-type proton ATPase 16 kDa proteolipid subunit | -0.473931188 |
| Q15370 | Transcription elongation factor B polypeptide 2 | -0.473931188 |
| Q969L2 | protein MAL2 | -0.473931188 |
| O43759-3 | Isoform 1C of Synaptogyrin-1 | -0.49410907 |
| P80723-2 | Isoform 2 of Brain acid soluble protein 1 | -0.49410907 |
| Q9NQC3-3 | Isoform 3 of Reticulon-4 | -0.514573173 |
| P01861 | Ig gamma-4 chain C region | -0.535331733 |
| P01009-1 | alpha-1-antitrypsin | -0.556393349 |
| Q13813-3 | Isoform 3 of Spectrin alpha chain, non-erythrocytic 1 | -0.556393349 |
| Q13885 | Tubulin beta-2A chain | -0.556393349 |
| P0CG38 | POTE ankyrin domain family member I | -0.577766999 |
| P0CG39 | POTE ankyrin domain family member J | -0.577766999 |
| Q6S8J3 | POTE ankyrin domain family member E | -0.577766999 |
| Q92686 | Neurogranin | -0.577766999 |
| Q9BYX7 | Putative beta-actin-like protein 3 | -0.577766999 |
| Q15833-1 | Syntaxin-binding protein 2 | -0.59946207 |
| P17677-1 | Neuromodulin | -0.621488377 |
| P17677-2 | Isoform 2 of Neuromodulin | -0.621488377 |
| Q9BS92 | Protein NipSnap homolog 3B | -0.621488377 |
| Q9BVA1 | Tubulin beta-2B chain | -0.621488377 |
| P19652 | Alpha-1-acid glycoprotein 2 | -0.785875195 |
| P02686-1 | myelin basic protein | -0.810966176 |
| Q05682-1 | Caldesmon | -0.971430848 |
| O60493-1 | sorting nexin-3 | -1 |
| O14983 | Sarcoplasmic/endoplasmic reticulum calcium ATPase 1 | -1.029146346 |
| O14983-2 | Isoform SERCA1A of Sarcoplasmic/endoplasmic reticulum calcium ATPase 1 | -1.029146346 |
| O14983-3 | Isoform 3 of Sarcoplasmic/endoplasmic reticulum calcium ATPase 1 | -1.029146346 |
| P20848 | Putative alpha-1-antitrypsin-related protein | -1.358453971 |

**Supplementary Table 7.** **Cortical proteins significantly altered in expression between the GRN p.0(IVS1+5G>C patients and multiple control individuals.** Primary iTRAQ expression ratios were Log_2_ transformed. Proteins represented in the table demonstrated a significant divergence in expression level (*P* <0.05) compared to the global mean protein expression level. For each significantly-regulated protein the associated Uniprot protein accession number, protein description and log_2_-transformed expression ratio are displayed.

| **Protein Accession** | **Protein Description** | **Log_2_ iTRAQ Ratio** |
| --- | --- | --- |
| P15259 | phosphoglycerate mutase 2 | 2.195347598 |
| P14136 | Glial fibrillary acidic protein | 1.545968369 |
| O00483 | Cytochrome c oxidase subunit NDUFA4 | 1.333423734 |
| Q9BX66-4 | Isoform 4 of Sorbin and SH3 domain-containing protein 1 | 1.117695043 |
| P20472 | Parvalbumin alpha | 1.111031312 |
| Q9BX66-1 | Sorbin and SH3 domain-containing protein 1 | 1.03562391 |
| Q6KB66 | Keratin, type II cytoskeletal 80 | 0.97819563 |
| Q6KB66-2 | Isoform 2 of Keratin, type II cytoskeletal 80 | 0.97819563 |
| O43298 | Zinc finger and BTB domain-containing protein 43 | 0.963474124 |
| P23142 | Fibulin-1 | 0.956056652 |
| P07108 | acyl-CoA-binding protein | 0.948600847 |
| Q13938-3 | Isoform 2 of Calcyphosin | 0.925999419 |
| Q14764 | major vault protein | 0.895302621 |
| Q02218 | 2-oxoglutarate dehydrogenase, mitochondrial | 0.879705766 |
| Q02218-2 | Isoform 2 of 2-oxoglutarate dehydrogenase, mitochondrial | 0.879705766 |
| P02792 | Ferritin light chain | 0.871843649 |
| Q86UD0 | Suppressor APC domain-containing protein 2 | 0.871843649 |
| Q2LD37-1 | Uncharacterized protein KIAA1109 | 0.86393845 |
| A8MUU1 | Putative fatty acid-binding protein 5-like protein 3 | 0.847996907 |
| P25713 | Metallothionein-3 | 0.847996907 |
| P17661 | desmin | 0.807354922 |
| Q01469 | Fatty acid-binding protein, epidermal | 0.799087306 |
| P29972-1 | aquaporin-1 | 0.790772038 |
| P50452 | Serpin B8 | 0.790772038 |
| P06703 | protein S100-A6 | 0.757023247 |
| Q5H9L2 | Transcription elongation factor A protein-like 5 | 0.757023247 |
| Q969E4 | transcription elongation factor A protein-like 3 | 0.757023247 |
| Q15942-2 | Isoform 2 of Zyxin | 0.748461233 |
| P36021 | Monocarboxylate transporter 8 | 0.739848103 |
| Q8N8S7 | Protein enabled homolog | 0.731183242 |
| Q9UII2-2 | Isoform 2 of ATPase inhibitor, mitochondrial | 0.731183242 |
| P07951-1 | Tropomyosin beta chain | 0.722466024 |
| O15173 | Membrane-associated progesterone receptor component 2 | 0.704871964 |
| P60201-2 | Isoform DM-20 of Myelin proteolipid protein | 0.695993813 |
| P00738 | Haptoglobin | 0.687060688 |
| Q02156 | Protein kinase C epsilon type | 0.687060688 |
| P01303 | Pro-neuropeptide Y | 0.678071905 |
| P17302 | Gap junction alpha-1 protein | 0.678071905 |
| P29966 | Myristoylated alanine-rich C-kinase substrate | 0.669026766 |
| P04179-3 | Isoform 3 of Superoxide dismutase [Mn], mitochondrial | 0.659924558 |
| P19013 | Keratin, type II cytoskeletal 4 | 0.659924558 |
| P30041 | Peroxiredoxin-6 | 0.659924558 |
| O43581-2 | Isoform 2 of Synaptotagmin-7 | 0.650764559 |
| Q13938 | Calcyphosin | 0.650764559 |
| P05787 | Keratin, type II cytoskeletal 8 | 0.641546029 |
| P05787-2 | Isoform 2 of Keratin, type II cytoskeletal 8 | 0.641546029 |
| P08729 | Keratin, type II cytoskeletal 7 | 0.641546029 |
| P05109 | Protein S100-A8 | 0.632268215 |
| P14621 | Acylphosphatase-2 | 0.632268215 |
| P50135 | Histamine N-methyltransferase | 0.632268215 |
| Q9UKU7 | Isobutyryl-CoA dehydrogenase, mitochondrial | 0.632268215 |
| P04179 | Superoxide dismutase [Mn], mitochondrial | 0.622930351 |
| P04179-4 | Isoform 4 of Superoxide dismutase [Mn], mitochondrial | 0.622930351 |
| P04271 | Protein S100-B | 0.613531653 |
| P60201-1 | Myelin proteolipid protein | 0.613531653 |
| Q8NFZ8 | Cell adhesion molecule 4 | 0.613531653 |
| Q9H444 | Charged multivesicular body protein 4b | 0.613531653 |
| P02689 | Myelin P2 protein | 0.604071324 |
| P27338 | Amine oxidase [flavin-containing] B | 0.604071324 |
| P46940 | Ras GTPase-activating-like protein IQGAP1 | 0.604071324 |
| P53985 | Monocarboxylate transporter 1 | 0.604071324 |
| P01861 | Ig gamma-4 chain C region | 0.59454855 |
| P02533 | Keratin, type I cytoskeletal 14 | 0.59454855 |
| P08779 | Keratin, type I cytoskeletal 16 | 0.59454855 |
| Q13162 | Peroxiredoxin-4 | 0.59454855 |
| O60234 | glia maturation factor gamma | 0.584962501 |
| P04179-2 | Isoform 2 of Superoxide dismutase [Mn], mitochondrial | 0.584962501 |
| P14866 | Heterogeneous nuclear ribonucleoprotein L | 0.584962501 |
| P14866-2 | Isoform 2 of Heterogeneous nuclear ribonucleoprotein L | 0.584962501 |
| P55060-2 | Isoform 2 of Exportin-2 | 0.584962501 |
| Q9ULV4 | coronin-1C | 0.584962501 |
| Q9Y6C9 | Mitochondrial carrier homolog 2 | 0.584962501 |
| P98160 | Basement membrane-specific heparan sulfate proteoglycan core protein | 0.575312331 |
| Q8N7J2-1 | APC membrane recruitment protein 2 | 0.575312331 |
| Q8N7J2-2 | Isoform 2 of APC membrane recruitment protein 2 | 0.575312331 |
| Q9H4A3 | Serine/threonine-protein kinase WNK1 | 0.575312331 |
| A6NMY6 | Putative annexin A2-like protein | 0.565597176 |
| P0DJI9 | Serum amyloid A-2 protein | 0.565597176 |
| P50897 | Palmitoyl-protein thioesterase 1 | 0.565597176 |
| Q96PE3-1 | Type I inositol 3,4-bisphosphate 4-phosphatase | 0.565597176 |
| O14745 | Na(+)/H(+) exchange regulatory cofactor NHE-RF1 | 0.555816155 |
| P62714 | serine/threonine-protein phosphatase 2A catalytic subunit beta isoform | 0.555816155 |
| Q9BW30 | Tubulin polymerization-promoting protein family member 3 | 0.555816155 |
| P09382 | Galectin-1 | 0.545968369 |
| P25311 | Zinc-alpha-2-glycoprotein | 0.545968369 |
| Q9UII2-1 | ATPase inhibitor, mitochondrial | 0.545968369 |
| O00168 | Phospholemman | 0.5360529 |
| O95837 | Guanine nucleotide-binding protein subunit alpha-14 | 0.5360529 |
| P01871-1 | Ig mu chain C region | 0.5360529 |
| P04220 | Ig MU heavy chain disease protein | 0.5360529 |
| P13611 | Versican core protein | 0.5360529 |
| Q8WY91 | THAP domain-containing protein 4 | 0.5360529 |
| Q9HC56 | Protocadherin-9 | 0.5360529 |
| Q9HC56-2 | Isoform 2 of Protocadherin-9 | 0.5360529 |
| P08758 | annexin A5 | 0.526068812 |
| P22570 | NADPH:adrenodoxin oxidoreductase, mitochondrial | 0.526068812 |
| P60900-3 | Isoform 3 of Proteasome subunit alpha type-6 | 0.526068812 |
| Q7KZF4 | staphylococcal nuclease domain-containing protein 1 | 0.526068812 |
| Q9ULH1-1 | Arf-GAP with SH3 domain, ANK repeat and PH domain-containing protein 1 | 0.526068812 |
| P04083 | annexin A1 | 0.516015147 |
| P08670 | Vimentin | 0.516015147 |
| P16070 | CD44 antigen | 0.516015147 |
| P20916-1 | Myelin-associated glycoprotein | 0.516015147 |
| P20916-2 | Isoform 2 of Myelin-associated glycoprotein | 0.516015147 |
| P00846 | ATP synthase subunit A | 0.50589093 |
| P50151 | Guanine nucleotide-binding protein G(I)/G(S)/G(O) subunit gamma-10 | 0.50589093 |
| Q15847 | Adipogenesis regulatory factor | 0.50589093 |
| Q9H0N5 | Pterin-4-alpha-carbinolamine dehydratase 2 | 0.50589093 |
| Q9H1X3-3 | Isoform 3 of DnaJ homolog subfamily C member 25 | 0.50589093 |
| Q9UQB3-1 | Catenin delta-2 | 0.50589093 |
| P24821-2 | Isoform 2 of Tenascin | 0.495695163 |
| Q14558 | Phosphoribosyl pyrophosphate synthase-associated protein 1 | 0.495695163 |
| Q14558-2 | Isoform 2 of Phosphoribosyl pyrophosphate synthase-associated protein 1 | 0.495695163 |
| Q15942 | Zyxin | 0.495695163 |
| Q96L46 | calpain small subunit 2 | 0.495695163 |
| Q9UPA5 | Protein bassoon | 0.495695163 |
| P55087-2 | Isoform 1 of Aquaporin-4 | 0.485426827 |
| P62244 | 40S ribosomal protein S15a | 0.485426827 |
| Q9H1E3 | Nuclear ubiquitous casein and cyclin-dependent kinase substrate 1 | 0.485426827 |
| Q9H1E3-2 | Isoform 2 of Nuclear ubiquitous casein and cyclin-dependent kinase substrate 1 | 0.485426827 |
| P37802 | Transgelin-2 | 0.475084883 |
| Q9NVH6-1 | Trimethyllysine dioxygenase, mitochondrial | 0.475084883 |
| P16083 | Ribosyldihydronicotinamide dehydrogenase [quinone] | 0.464668267 |
| Q9GZV7 | Hyaluronan and proteoglycan link protein 2 | 0.464668267 |
| P55087 | Aquaporin-4 | 0.454175893 |
| Q9UMX0-1 | Ubiquilin-1 | 0.432959407 |
| P41219 | Peripherin | 0.422233001 |
| P02008 | Hemoglobin subunit zeta | 0.378511623 |
| P13473-1 | Lysosome-associated membrane glycoprotein 2 | 0.378511623 |
| O00499-8 | Isoform BIN1 of Myc box-dependent-interacting protein 1 | 0.367371066 |
| P31689-1 | DnaJ homolog subfamily A member 1 | 0.367371066 |
| Q08495-1 | Dematin | 0.367371066 |
| O43598-1 | 2'-deoxynucleoside 5'-phosphate N-hydrolase 1 | 0.35614381 |
| O43598-2 | Isoform 2 of 2'-deoxynucleoside 5'-phosphate N-hydrolase 1 | 0.35614381 |
| P02511 | Alpha-crystallin B chain | 0.35614381 |
| P49189 | 4-trimethylaminobutyraldehyde dehydrogenase | 0.35614381 |
| P49419-3 | Isoform 3 of Alpha-aminoadipic semialdehyde dehydrogenase | 0.35614381 |
| P57058 | Hormonally up-regulated neu tumor-associated kinase | 0.35614381 |
| Q8IXS6 | paralemmin-2 | 0.35614381 |
| P02538 | Keratin, type II cytoskeletal 6A | 0.344828497 |
| P48668 | Keratin, type II cytoskeletal 6C | 0.344828497 |
| Q01546 | Keratin, type II cytoskeletal 2 oral | 0.344828497 |
| P00918 | Carbonic anhydrase 2 | 0.333423734 |
| Q06830 | peroxiredoxin-1 | 0.333423734 |
| Q14CZ8-1 | Hepatocyte cell adhesion molecule | 0.333423734 |
| Q9Y2J8-2 | Isoform 2 of Protein-arginine deiminase type-2 | 0.333423734 |
| Q9Y2S2-1 | Lambda-crystallin homolog | 0.333423734 |
| Q9Y2S2-2 | Isoform 2 of Lambda-crystallin homolog | 0.333423734 |
| Q9Y617-1 | phosphoserine aminotransferase | 0.333423734 |
| P0DJI8 | Serum amyloid A-1 protein | 0.321928095 |
| P21926 | CD9 antigen | 0.321928095 |
| P35749 | Myosin-11 | 0.321928095 |
| Q13424-2 | Isoform 2 of Alpha-1-syntrophin | 0.321928095 |
| Q14CZ8-2 | Isoform 2 of Hepatocyte cell adhesion molecule | 0.321928095 |
| P54819-6 | Isoform 6 of Adenylate kinase 2, mitochondrial | 0.310340121 |
| P55263-1 | Adenosine kinase | 0.310340121 |
| Q13336 | urea transporter 1 | 0.310340121 |
| Q13336-2 | Isoform 2 of Urea transporter 1 | 0.310340121 |
| Q9Y2J8 | Protein-arginine deiminase type-2 | 0.310340121 |
| O75828 | Carbonyl reductase [NADPH] 3 | 0.298658316 |
| P26447 | Protein S100-A4 | 0.286881148 |
| Q9Y2V2 | Calcium-regulated heat stable protein 1 | 0.286881148 |
| P61457 | Pterin-4-alpha-carbinolamine dehydratase | 0.275007047 |
| Q96FQ6 | Protein S100-A16 | 0.275007047 |
| P19022 | Cadherin-2 | 0.263034406 |
| P07355 | Annexin A2 | 0.250961574 |
| Q3KQU3-3 | Isoform 3 of MAP7 domain-containing protein 1 | 0.250961574 |
| Q9NZ01-1 | Very-long-chain enoyl-CoA reductase | 0.250961574 |
| P15311 | Ezrin | 0.23878686 |
| P41222 | Prostaglandin-H2 D-isomerase | 0.23878686 |
| Q3SY69-1 | Mitochondrial 10-formyltetrahydrofolate dehydrogenase | 0.22650853 |
| Q8NGA1 | Olfactory receptor 1M1 | 0.22650853 |
| P00568 | Adenylate kinase isoenzyme 1 | 0.214124805 |
| P11279 | Lysosome-associated membrane glycoprotein 1 | 0.214124805 |
| P49189-2 | Isoform 2 of 4-trimethylaminobutyraldehyde dehydrogenase | 0.214124805 |
| P49419 | Alpha-aminoadipic semialdehyde dehydrogenase | 0.214124805 |
| Q5SSJ5-1 | Heterochromatin protein 1-binding protein 3 | 0.214124805 |
| Q92769 | Histone deacetylase 2 | 0.214124805 |
| P21695-1 | Glycerol-3-phosphate dehydrogenase [NAD(+)], cytoplasmic | 0.201633861 |
| P35080-1 | Profilin-2 | -0.32192809 |
| Q7Z3Y7 | keratin, type I cytoskeletal 28 | -0.32192809 |
| Q7Z3Y8 | Keratin, type I cytoskeletal 27 | -0.32192809 |
| Q7Z3Z0 | Keratin, type I cytoskeletal 25 | -0.32192809 |
| Q9BXW6-1 | Oxysterol-binding protein-related protein 1 | -0.32192809 |
| Q9BXW6-2 | Isoform A of Oxysterol-binding protein-related protein 1 | -0.32192809 |
| P26640 | Valine--tRNA ligase | -0.35845397 |
| P35527 | Keratin, type I cytoskeletal 9 | -0.35845397 |
| Q15008 | 26S proteasome non-ATPase regulatory subunit 6 | -0.35845397 |
| Q15008-4 | Isoform 4 of 26S proteasome non-ATPase regulatory subunit 6 | -0.35845397 |
| Q9BWS9-1 | Chitinase domain-containing protein 1 | -0.35845397 |
| P25685 | dnaJ homolog subfamily B member 1 | -0.39592868 |
| Q8TDB8-3 | Isoform 3 of Solute carrier family 2, facilitated glucose transporter member 14 | -0.39592868 |
| P01034 | Cystatin-C | -0.4150375 |
| Q15008-2 | Isoform 2 of 26S proteasome non-ATPase regulatory subunit 6 | -0.43440282 |
| P04264 | Keratin, type II cytoskeletal 1 | -0.45403163 |
| P04350 | Tubulin beta-4A chain | -0.47393119 |
| P25098 | Beta-adrenergic receptor kinase 1 | -0.47393119 |
| Q6U841 | Sodium-driven chloride bicarbonate exchanger | -0.47393119 |
| Q8NC96 | adaptin ear-binding coat-associated protein 1 | -0.49410907 |
| Q9H1N7-1 | Adenosine 3'-phospho 5'-phosphosulfate transporter 2 | -0.49410907 |
| Q04917 | 14-3-3 protein eta | -0.53533173 |
| Q9H4M9 | EH domain-containing protein 1 | -0.53533173 |
| A2BFH1 | peptidyl-prolyl cis-trans isomerase A-like 4G | -0.55639335 |
| F5H284 | peptidyl-prolyl cis-trans isomerase A-like 4D | -0.55639335 |
| P49821 | NADH dehydrogenase [ubiquinone] flavoprotein 1, mitochondrial | -0.55639335 |
| P63215 | Guanine nucleotide-binding protein G(I)/G(S)/G(O) subunit gamma-3 | -0.55639335 |
| O00629 | Importin subunit alpha-3 | -0.577767 |
| Q15233 | Non-POU domain-containing octamer-binding protein | -0.577767 |
| Q9P2R7 | Succinyl-CoA ligase [ADP-forming] subunit beta, mitochondrial | -0.577767 |
| Q58FF8 | Putative heat shock protein HSP 90-beta 2 | -0.59946207 |
| Q5K651 | Sterile alpha motif domain-containing protein 9 | -0.59946207 |
| Q8IVG5 | sterile alpha motif domain-containing protein 9-like | -0.59946207 |
| P09496-5 | Isoform 5 of Clathrin light chain A | -0.62148838 |
| P30419 | glycylpeptide N-tetradecanoyltransferase 1 | -0.62148838 |
| Q9UHD1 | cysteine and histidine-rich domain-containing protein 1 | -0.62148838 |
| P49758-4 | regulator of G-protein signaling 6 | -0.64385619 |
| P01877 | Ig alpha-2 chain C region | -0.68965988 |
| P61966 | AP-1 complex subunit sigma-1A | -0.68965988 |
| P61966-2 | Isoform 2 of AP-1 complex subunit sigma-1A | -0.68965988 |
| P09936 | Ubiquitin carboxyl-terminal hydrolase isozyme L1 | -0.71311885 |
| P46783 | 40S ribosomal protein S10 | -0.71311885 |
| P61764 | Syntaxin-binding protein 1 | -0.71311885 |
| Q15818 | Neuronal pentraxin-1 | -0.71311885 |
| Q9UQM7-2 | Isoform B of Calcium/calmodulin-dependent protein kinase type II subunit alpha | -0.71311885 |
| O14994 | Synapsin-3 | -0.73696559 |
| P05155 | Plasma protease C1 inhibitor | -0.73696559 |
| P68366 | Tubulin alpha-4A chain | -0.73696559 |
| P68366-2 | Isoform 2 of Tubulin alpha-4A chain | -0.73696559 |
| Q06055 | ATP synthase F(0) complex subunit C2, mitochondrial | -0.73696559 |
| Q7L1I2-2 | Isoform 2 of Synaptic vesicle glycoprotein 2B | -0.73696559 |
| Q99622 | Protein C10 | -0.73696559 |
| Q9NPY3 | Complement component C1q receptor | -0.73696559 |
| Q9P2U7-2 | Isoform 2 of Vesicular glutamate transporter 1 | -0.73696559 |
| Q9UJZ1 | Stomatin-like protein 2, mitochondrial | -0.73696559 |
| Q9Y487 | V-type proton ATPase 116 kDa subunit a isoform 2 | -0.73696559 |
| P11234 | Ras-related protein Ral-B | -0.76121314 |
| P13591-6 | Isoform 6 of Neural cell adhesion molecule 1 | -0.76121314 |
| P49802-1 | regulator of G-protein signaling 7 | -0.76121314 |
| Q07021 | Complement component 1 Q subcomponent-binding protein, mitochondrial | -0.76121314 |
| O95670-2 | Isoform 2 of V-type proton ATPase subunit G 2 | -0.78587519 |
| P01859 | Ig gamma-2 chain C region | -0.78587519 |
| P08754 | Guanine nucleotide-binding protein G(k) subunit alpha | -0.78587519 |
| P49069 | calcium signal-modulating cyclophilin ligand | -0.78587519 |
| P49756-1 | RNA-binding protein 25 | -0.78587519 |
| P61328-1 | Fibroblast growth factor 12 | -0.78587519 |
| Q7L1I2 | Synaptic vesicle glycoprotein 2B | -0.78587519 |
| Q86Y22-1 | Collagen alpha-1(XXIII) chain | -0.78587519 |
| Q99719-1 | Septin-5 | -0.78587519 |
| Q9UD71-2 | Isoform 2 of Protein phosphatase 1 regulatory subunit 1B | -0.78587519 |
| P62249 | 40S ribosomal protein S16 | -0.81096618 |
| Q16718 | NADH dehydrogenase [ubiquinone] 1 alpha subcomplex subunit 5 | -0.81096618 |
| Q58FG0 | Putative heat shock protein HSP 90-alpha A5 | -0.81096618 |
| Q9Y570 | Protein phosphatase methylesterase 1 | -0.81096618 |
| P16104 | Histone H2AX | -0.83650127 |
| P61981 | 14-3-3 protein gamma | -0.83650127 |
| Q13885 | Tubulin beta-2A chain | -0.83650127 |
| Q8IUE6 | Histone H2A type 2-B | -0.83650127 |
| Q96QV6 | Histone H2A type 1-A | -0.83650127 |
| Q99729-1 | Heterogeneous nuclear ribonucleoprotein A/B | -0.83650127 |
| Q9Y639-2 | Neuroplastin | -0.83650127 |
| A6NNZ2 | Tubulin beta-8 chain-like protein LOC260334 | -0.86249648 |
| P01009-1 | alpha-1-antitrypsin | -0.86249648 |
| P61328-2 | Isoform 2 of Fibroblast growth factor 12 | -0.86249648 |
| Q13813-3 | Isoform 3 of Spectrin alpha chain, non-erythrocytic 1 | -0.86249648 |
| Q3ZCM7 | tubulin beta-8 chain | -0.86249648 |
| Q92915 | fibroblast growth factor 14 | -0.86249648 |
| Q969L2 | protein MAL2 | -0.86249648 |
| Q9BVA1 | Tubulin beta-2B chain | -0.86249648 |
| Q9NQC3-3 | Isoform 3 of Reticulon-4 | -0.86249648 |
| P19652 | Alpha-1-acid glycoprotein 2 | -0.88896869 |
| Q9GZN7 | Protein rogdi homolog | -0.88896869 |
| Q9Y639-1 | Isoform 1 of Neuroplastin | -0.88896869 |
| P17677-1 | Neuromodulin | -0.91593574 |
| Q92686 | Neurogranin | -0.91593574 |
| P01009-2 | Isoform 2 of Alpha-1-antitrypsin | -0.97143085 |
| P02686-1 | myelin basic protein | -0.97143085 |
| P08247 | Synaptophysin | -0.97143085 |
| P27449 | V-type proton ATPase 16 kDa proteolipid subunit | -0.97143085 |
| Q15904 | V-type proton ATPase subunit S1 | -0.97143085 |
| Q9BS92 | Protein NipSnap homolog 3B | -0.97143085 |
| P08247-2 | Isoform 2 of Synaptophysin | -1 |
| Q8N196 | homeobox protein SIX5 | -1 |
| P0CG38 | POTE ankyrin domain family member I | -1.02914635 |
| P0CG39 | POTE ankyrin domain family member J | -1.02914635 |
| P80723 | Brain acid soluble protein 1 | -1.02914635 |
| Q6S8J3 | POTE ankyrin domain family member E | -1.02914635 |
| Q9BYX7 | Putative beta-actin-like protein 3 | -1.02914635 |
| P80723-2 | Isoform 2 of Brain acid soluble protein 1 | -1.05889369 |
| Q05682-1 | Caldesmon | -1.12029423 |
| Q05682-2 | Isoform 2 of Caldesmon | -1.12029423 |
| O60493-1 | sorting nexin-3 | -1.25153877 |
| O43759-3 | Isoform 1C of Synaptogyrin-1 | -1.32192809 |
| Q15833-1 | Syntaxin-binding protein 2 | -1.43440282 |
| P20848 | Putative alpha-1-antitrypsin-related protein | -1.68965988 |
| O14983 | Sarcoplasmic/endoplasmic reticulum calcium ATPase 1 | -2.18442457 |

**Supplementary Table 8.** **Cortical proteins significantly altered in expression between the *VCP* p.R159H patients and multiple control individuals**. Primary iTRAQ expression ratios were Log_2_ transformed. Proteins represented in the table demonstrated a significant divergence in expression level (*P* <0.05) compared to the global protein expression level. For each significantly-regulated protein the associated Uniprot protein accession number, protein description and log_2_-transformed expression ratio are displayed.

| **Protein Accession** | **Protein Description** | **Log_2_ iTRAQ Ratio** |
| --- | --- | --- |
| Q15942-2 | Isoform 2 of Zyxin | 2.375734539 |
| Q9UQB3-1 | Catenin delta-2 | 1.871843649 |
| Q16720-1 | Plasma membrane calcium-transporting ATPase 3 | 1.422233001 |
| Q15942 | Zyxin | 1.182692298 |
| P42356-1 | phosphatidylinositol 4-kinase alpha | 1.14404637 |
| P02008 | Hemoglobin subunit zeta | 1.03562391 |
| P15259 | phosphoglycerate mutase 2 | 1.021479727 |
| P00738 | Haptoglobin | 0.90303827 |
| P00739-1 | Haptoglobin-related protein | 0.887525271 |
| P49758-4 | regulator of G-protein signaling 6 | 0.815575429 |
| A8MUU1 | Putative fatty acid-binding protein 5-like protein 3 | 0.799087306 |
| Q5TC84 | Opioid growth factor receptor-like protein 1 | 0.790772038 |
| Q8WZA0 | Protein LZIC | 0.790772038 |
| P30039 | Phenazine biosynthesis-like domain-containing protein | 0.782408565 |
| Q9Y276 | Mitochondrial chaperone BCS1 | 0.782408565 |
| O95674-1 | phosphatidate cytidylyltransferase 2 | 0.773996325 |
| P69905 | Hemoglobin subunit alpha | 0.731183242 |
| P62081 | 40S ribosomal protein S7 | 0.713695815 |
| Q9NQX3 | Gephyrin | 0.704871964 |
| Q08257-2 | Isoform 2 of Quinone oxidoreductase | 0.695993813 |
| P09488 | Glutathione S-transferase Mu 1 | 0.687060688 |
| P09488-2 | Isoform 2 of Glutathione S-transferase Mu 1 | 0.687060688 |
| Q03013-1 | glutathione S-transferase mu 4 | 0.687060688 |
| Q03013-2 | Isoform 2 of Glutathione S-transferase Mu 4 | 0.687060688 |
| Q9ULV4 | coronin-1C | 0.678071905 |
| P01861 | Ig gamma-4 chain C region | 0.669026766 |
| Q8TB36 | ganglioside-induced differentiation-associated protein 1 | 0.659924558 |
| Q9UHD1 | cysteine and histidine-rich domain-containing protein 1 | 0.641546029 |
| A6NCE7 | Microtubule-associated proteins 1A/1B light chain 3 beta 2 | 0.622930351 |
| P03915 | NADH-ubiquinone oxidoreductase chain 5 | 0.622930351 |
| P68871 | Hemoglobin subunit beta | 0.622930351 |
| Q9GZQ8 | Microtubule-associated proteins 1A/1B light chain 3B | 0.622930351 |
| O00468 | Agrin | 0.613531653 |
| P05109 | Protein S100-A8 | 0.613531653 |
| P50416 | Carnitine O-palmitoyltransferase 1, liver isoform | 0.613531653 |
| P57058 | Hormonally up-regulated neu tumor-associated kinase | 0.613531653 |
| Q5T619 | Zinc finger protein 648 | 0.613531653 |
| P02730 | Band 3 anion transport protein | 0.604071324 |
| Q08431 | Lactadherin | 0.604071324 |
| P35749 | Myosin-11 | 0.59454855 |
| P62714 | serine/threonine-protein phosphatase 2A catalytic subunit beta isoform | 0.59454855 |
| Q9HC56 | Protocadherin-9 | 0.59454855 |
| O14976 | Cyclin-G-associated kinase | 0.584962501 |
| P62304 | small nuclear ribonucleoprotein E | 0.584962501 |
| P35580 | Myosin-10 | 0.575312331 |
| P46940 | Ras GTPase-activating-like protein IQGAP1 | 0.575312331 |
| Q5TCY1-1 | Tau-tubulin kinase 1 | 0.575312331 |
| Q9Y5Z4-1 | Heme-binding protein 2 | 0.575312331 |
| P01871-1 | Ig mu chain C region | 0.565597176 |
| P04220 | Ig MU heavy chain disease protein | 0.565597176 |
| P06702 | Protein S100-A9 | 0.565597176 |
| P08754 | Guanine nucleotide-binding protein G(k) subunit alpha | 0.565597176 |
| P14136 | Glial fibrillary acidic protein | 0.565597176 |
| P20774 | Mimecan | 0.565597176 |
| Q96PE3-1 | Type I inositol 3,4-bisphosphate 4-phosphatase | 0.565597176 |
| O95139 | NADH dehydrogenase [ubiquinone] 1 beta subcomplex subunit 6 | 0.555816155 |
| Q02218 | 2-oxoglutarate dehydrogenase, mitochondrial | 0.545968369 |
| Q86UD0 | Suppressor APC domain-containing protein 2 | 0.545968369 |
| Q8TAC9-1 | Secretory carrier-associated membrane protein 5 | 0.545968369 |
| P08670 | Vimentin | 0.5360529 |
| P42126 | Enoyl-CoA delta isomerase 1, mitochondrial | 0.5360529 |
| Q9H845 | Acyl-CoA dehydrogenase family member 9, mitochondrial | 0.5360529 |
| P02042 | Hemoglobin subunit delta | 0.526068812 |
| P17661 | desmin | 0.526068812 |
| Q15334 | Lethal(2) giant larvae protein homolog 1 | 0.526068812 |
| P0DJI9 | Serum amyloid A-2 protein | 0.516015147 |
| P0DJI9-2 | Isoform 2 of Serum amyloid A-2 protein | 0.516015147 |
| Q01469 | Fatty acid-binding protein, epidermal | 0.516015147 |
| Q15008-2 | Isoform 2 of 26S proteasome non-ATPase regulatory subunit 6 | 0.516015147 |
| Q15811 | Intersectin-1 | 0.516015147 |
| O14735 | CDP-diacylglycerol--inositol 3-phosphatidyltransferase | 0.516015147 |
| Q3SY69-1 | Mitochondrial 10-formyltetrahydrofolate dehydrogenase | 0.516015147 |
| P17066 | Heat shock 70 kDa protein 6 | 0.50589093 |
| Q92833 | Protein Jumonji | 0.50589093 |
| Q3SY69-1 | Mitochondrial 10-formyltetrahydrofolate dehydrogenase | 0.495695163 |
| P41222 | Prostaglandin-H2 D-isomerase | 0.475084883 |
| Q2LD37-1 | Uncharacterized protein KIAA1109 | 0.432959407 |
| O43298 | Zinc finger and BTB domain-containing protein 43 | 0.422233001 |
| P29966 | Myristoylated alanine-rich C-kinase substrate | 0.422233001 |
| P61020-2 | Isoform 2 of Ras-related protein Rab-5B | 0.411426246 |
| P00915 | carbonic anhydrase 1 | 0.40053793 |
| P36021 | Monocarboxylate transporter 8 | 0.40053793 |
| P62714 | serine/threonine-protein phosphatase 2A catalytic subunit beta isoform | 0.40053793 |
| P29762 | Cellular retinoic acid-binding protein 1 | 0.389566812 |
| P45985 | Dual specificity mitogen-activated protein kinase kinase 4 | 0.389566812 |
| P50135 | Histamine N-methyltransferase | 0.389566812 |
| Q15008 | 26S proteasome non-ATPase regulatory subunit 6 | 0.389566812 |
| P05771-1 | Protein kinase C beta type | 0.367371066 |
| P07108 | acyl-CoA-binding protein | 0.367371066 |
| P31689-1 | DnaJ homolog subfamily A member 1 | 0.367371066 |
| Q14558 | Phosphoribosyl pyrophosphate synthase-associated protein 1 | 0.367371066 |
| Q14558-2 | Isoform 2 of Phosphoribosyl pyrophosphate synthase-associated protein 1 | 0.367371066 |
| Q8N8S7 | Protein enabled homolog | 0.344828497 |
| Q96BJ3-1 | Axin interactor, dorsalization-associated protein | 0.344828497 |
| P30711 | Glutathione S-transferase theta-1 | 0.333423734 |
| Q9BSJ8 | Extended synaptotagmin-1 | 0.333423734 |
| P35219 | carbonic anhydrase-related protein | 0.310340121 |
| O43143 | Putative pre-mRNA-splicing factor ATP-dependent RNA helicase DHX15 | 0.298658316 |
| P19971 | thymidine phosphorylase | 0.298658316 |
| P05787 | Keratin, type II cytoskeletal 8 | 0.275007047 |
| P08729 | Keratin, type II cytoskeletal 7 | 0.275007047 |
| P11279 | Lysosome-associated membrane glycoprotein 1 | 0.275007047 |
| P42025 | Beta-centractin | 0.275007047 |
| Q86TV6-1 | Tetratricopeptide repeat protein 7B | 0.275007047 |
| Q9H0N5 | Pterin-4-alpha-carbinolamine dehydratase 2 | 0.275007047 |
| P08571 | Monocyte differentiation antigen CD14 | 0.263034406 |
| P41219 | Peripherin | 0.263034406 |
| P41219-2 | Isoform 2 of Peripherin | 0.263034406 |
| Q9NZ01-1 | Very-long-chain enoyl-CoA reductase | 0.263034406 |
| P07951-1 | Tropomyosin beta chain | 0.250961574 |
| P30041 | Peroxiredoxin-6 | 0.250961574 |
| Q96J87-1 | CUGBP Elav-like family member 6 | 0.23878686 |
| Q9H1E3 | Nuclear ubiquitous casein and cyclin-dependent kinase substrate 1 | 0.23878686 |
| A6NMY6 | Putative annexin A2-like protein | 0.22650853 |
| Q14764 | major vault protein | 0.22650853 |
| Q9UD71-2 | Isoform 2 of Protein phosphatase 1 regulatory subunit 1B | 0.22650853 |
| P13611 | Versican core protein | 0.201633861 |
| P61328-1 | Fibroblast growth factor 12 | 0.201633861 |
| Q92915 | fibroblast growth factor 14 | 0.201633861 |
| Q99729-1 | Heterogeneous nuclear ribonucleoprotein A/B | 0.201633861 |
| P12081 | Histidine--tRNA ligase, cytoplasmic | 0.189033824 |
| P24821-2 | Isoform 2 of Tenascin | 0.189033824 |
| Q99598 | Translin-associated protein X | 0.189033824 |
| P0CG29 | Glutathione S-transferase theta-2 | 0.176322773 |
| P13726-1 | tissue factor | 0.176322773 |
| Q8N196 | homeobox protein SIX5 | 0.176322773 |
| Q9P265 | Disco-interacting protein 2 homolog B | 0.176322773 |
| O43681 | ATPase ASNA1 | 0.163498732 |
| P02768-1 | Serum albumin | 0.163498732 |
| Q13162 | Peroxiredoxin-4 | 0.163498732 |
| Q8TCD5-2 | Isoform 2 of 5'(3')-deoxyribonucleotidase, cytosolic type | 0.163498732 |
| Q8WY91 | THAP domain-containing protein 4 | 0.163498732 |
| Q9BW30 | Tubulin polymerization-promoting protein family member 3 | 0.163498732 |
| Q9NVH6-1 | Trimethyllysine dioxygenase, mitochondrial | 0.163498732 |
| Q9NZ56 | Formin-2 | 0.163498732 |
| P0C0L4-1 | Complement C4-A | 0.124328135 |
| P20916-1 | Myelin-associated glycoprotein | 0.124328135 |
| Q9ULH1-1 | Arf-GAP with SH3 domain, ANK repeat and PH domain-containing protein 1 | 0.111031312 |
| P68400 | Casein kinase II subunit alpha | 0.084064265 |
| Q8NEV1 | Casein kinase II subunit alpha 3 | 0.084064265 |
| P04271 | Protein S100-B | 0.056583528 |
| Q17R31-1 | Putative deoxyribonuclease TATDN3 | 0.056583528 |
| O43633 | charged multivesicular body protein 2a | 0.042644337 |
| P14618 | Pyruvate kinase PKM | 0.042644337 |
| P28161 | Glutathione S-transferase Mu 2 | 0.042644337 |
| P42330 | Aldo-keto reductase family 1 member C3 | 0.028569152 |
| P50452 | Serpin B8 | 0.028569152 |
| O95248-1 | Myotubularin-related protein 5 | 0.014355293 |
| P01877 | Ig alpha-2 chain C region | 0 |
| P23297 | Protein S100-A1 | 0 |
| Q9BWS9-1 | Chitinase domain-containing protein 1 | 0 |
| Q9GZV7 | Hyaluronan and proteoglycan link protein 2 | 0 |
| O15067 | Phosphoribosylformylglycinamidine synthase | -0.01449957 |
| O75369-1 | Filamin-B | -0.01449957 |
| P56385 | ATP synthase subunit e, mitochondrial | -0.01449957 |
| Q8NGA1 | Olfactory receptor 1M1 | -0.029146346 |
| Q9Y2V2 | Calcium-regulated heat stable protein 1 | -0.029146346 |
| Q9UMX0-1 | Ubiquilin-1 | -0.043943348 |
| P07355 | Annexin A2 | -0.058893689 |
| P08758 | annexin A5 | -0.058893689 |
| Q12906-1 | Interleukin enhancer-binding factor 3 | -0.058893689 |
| Q14254 | Flotillin-2 | -0.058893689 |
| P02689 | Myelin P2 protein | -0.358453971 |
| Q16653-5 | Isoform 5 of Myelin-oligodendrocyte glycoprotein | -0.358453971 |
| Q08257-2 | Isoform 2 of Quinone oxidoreductase | -0.395928676 |
| Q96HY6-1 | DDRGK domain-containing protein 1 | -0.395928676 |
| P60201-1 | Myelin proteolipid protein | -0.454031631 |
| P01034 | Cystatin-C | -0.514573173 |
| P07197-2 | Isoform 2 of Neurofilament medium polypeptide | -0.514573173 |
| P09496-5 | Isoform 5 of Clathrin light chain A | -0.514573173 |
| P48556 | 26S proteasome non-ATPase regulatory subunit 8 | -0.514573173 |
| Q8TAM6 | ermin | -0.514573173 |
| Q8WYK1 | Contactin-associated protein-like 5 | -0.514573173 |
| Q9UM22-2 | Isoform 2 of Mammalian ependymin-related protein 1 | -0.514573173 |
| O95197-5 | Isoform 5 of Reticulon-3 | -0.535331733 |
| P01112-2 | Isoform 2 of GTPase HRas | -0.577766999 |
| Q7Z6L0-3 | Isoform 3 of Proline-rich transmembrane protein 2 | -0.577766999 |
| Q9UII2-2 | Isoform 2 of ATPase inhibitor, mitochondrial | -0.577766999 |
| P07197 | Neurofilament medium polypeptide | -0.59946207 |
| P12036 | Neurofilament heavy polypeptide | -0.59946207 |
| Q5K651 | Sterile alpha motif domain-containing protein 9 | -0.59946207 |
| Q8IVG5 | sterile alpha motif domain-containing protein 9-like | -0.59946207 |
| Q9BWQ8 | protein lifeguard 2 | -0.59946207 |
| P04156-1 | Major prion protein | -0.621488377 |
| P68371 | Tubulin beta-4B chain | -0.621488377 |
| P07196 | Neurofilament light polypeptide | -0.64385619 |
| P20290-1 | transcription factor BTF3 | -0.64385619 |
| P43004-1 | excitatory amino acid transporter 2 | -0.64385619 |
| P49750-1 | YLP motif-containing protein 1 | -0.64385619 |
| Q5TBK1-1 | NEDD4-binding protein 2-like 1 | -0.64385619 |
| Q7Z6L0-1 | Proline-rich transmembrane protein 2 | -0.64385619 |
| Q86UW6 | NEDD4-binding protein 2 | -0.64385619 |
| Q92802-1 | NEDD4-binding protein 2-like 2 | -0.64385619 |
| P00338-1 | L-lactate dehydrogenase A chain | -0.666576266 |
| P05496 | ATP synthase F(0) complex subunit C1, mitochondrial | -0.666576266 |
| P06753 | Tropomyosin alpha-3 chain | -0.666576266 |
| P48201 | ATP synthase F(0) complex subunit C3, mitochondrial | -0.666576266 |
| P60201-2 | Isoform DM-20 of Myelin proteolipid protein | -0.666576266 |
| Q06055 | ATP synthase F(0) complex subunit C2, mitochondrial | -0.666576266 |
| Q06055-2 | Isoform 2 of ATP synthase F(0) complex subunit C2, mitochondrial | -0.666576266 |
| Q92686 | Neurogranin | -0.666576266 |
| P62328 | Thymosin beta-4 | -0.689659879 |
| P63313 | thymosin beta-10 | -0.689659879 |
| Q13813 | Spectrin alpha chain, non-erythrocytic 1 | -0.689659879 |
| P19013 | Keratin, type II cytoskeletal 4 | -0.713118852 |
| Q2M2I8-2 | Isoform 2 of AP2-associated protein kinase 1 | -0.713118852 |
| A6NNZ2 | Tubulin beta-8 chain-like protein LOC260334 | -0.736965594 |
| O60262 | guanine nucleotide-binding protein G(I)/G(S)/G(O) subunit gamma-7 | -0.736965594 |
| O95294-1 | RasGAP-activating-like protein 1 | -0.736965594 |
| P02538 | Keratin, type II cytoskeletal 6A | -0.736965594 |
| P04259 | keratin, type II cytoskeletal 6B | -0.736965594 |
| P04264 | Keratin, type II cytoskeletal 1 | -0.736965594 |
| P13645 | Keratin, type I cytoskeletal 10 | -0.736965594 |
| P35908 | Keratin, type II cytoskeletal 2 epidermal | -0.736965594 |
| P48668 | Keratin, type II cytoskeletal 6C | -0.736965594 |
| P49069 | calcium signal-modulating cyclophilin ligand | -0.736965594 |
| P61764 | Syntaxin-binding protein 1 | -0.736965594 |
| Q01546 | Keratin, type II cytoskeletal 2 oral | -0.736965594 |
| Q08211-2 | Isoform 2 of ATP-dependent RNA helicase A | -0.736965594 |
| Q08722 | Leukocyte surface antigen CD47 | -0.736965594 |
| Q3ZCM7 | tubulin beta-8 chain | -0.736965594 |
| Q6NXT2 | Histone H3.3C | -0.736965594 |
| Q7Z3Y7 | keratin, type I cytoskeletal 28 | -0.736965594 |
| Q7Z3Z0 | Keratin, type I cytoskeletal 25 | -0.736965594 |
| Q9NQC3-3 | Isoform 3 of Reticulon-4 | -0.736965594 |
| Q9UJ78-4 | Zinc finger MYM-type protein 5 | -0.736965594 |
| Q9Y2J2-2 | Isoform 2 of Band 4.1-like protein 3 | -0.736965594 |
| O43759-1 | Synaptogyrin-1 | -0.76121314 |
| P05026 | Sodium/potassium-transporting ATPase subunit beta-1 | -0.76121314 |
| P05026-2 | Isoform 2 of Sodium/potassium-transporting ATPase subunit beta-1 | -0.76121314 |
| Q58FF8 | Putative heat shock protein HSP 90-beta 2 | -0.76121314 |
| Q58FG1 | Putative heat shock protein HSP 90-alpha A4 | -0.76121314 |
| Q86Y22-1 | Collagen alpha-1(XXIII) chain | -0.76121314 |
| Q93008-1 | Isoform 2 of Probable ubiquitin carboxyl-terminal hydrolase FAF-X | -0.76121314 |
| O95299 | NADH dehydrogenase [ubiquinone] 1 alpha subcomplex subunit 10, mitochondrial | -0.785875195 |
| P30419 | glycylpeptide N-tetradecanoyltransferase 1 | -0.785875195 |
| P30419-2 | Isoform Short of Glycylpeptide N-tetradecanoyltransferase 1 | -0.785875195 |
| P35080-1 | Profilin-2 | -0.785875195 |
| P35527 | Keratin, type I cytoskeletal 9 | -0.785875195 |
| P35609 | Alpha-actinin-2 | -0.785875195 |
| P36542-1 | ATP synthase subunit gamma, mitochondrial | -0.785875195 |
| P80723 | Brain acid soluble protein 1 | -0.785875195 |
| Q9Y639-2 | Neuroplastin | -0.785875195 |
| P01009-1 | alpha-1-antitrypsin | -0.810966176 |
| P20929 | Nebulin | -0.810966176 |
| P80723-2 | Isoform 2 of Brain acid soluble protein 1 | -0.810966176 |
| Q08495-1 | Dematin | -0.810966176 |
| Q13885 | Tubulin beta-2A chain | -0.810966176 |
| Q99726 | Zinc transporter 3 | -0.810966176 |
| Q9BVA1 | Tubulin beta-2B chain | -0.810966176 |
| Q9H305 | Cell death-inducing p53-target protein 1 | -0.810966176 |
| Q9NZ45 | CDGSH iron-sulfur domain-containing protein 1 | -0.810966176 |
| Q9NZQ3-1 | NCK-interacting protein with SH3 domain | -0.810966176 |
| Q9UQN3-1 | Charged multivesicular body protein 2b | -0.810966176 |
| O14810 | Complexin-1 | -0.836501268 |
| P01009-3 | Isoform 3 of Alpha-1-antitrypsin | -0.836501268 |
| P05129 | Protein kinase C gamma type | -0.836501268 |
| P05129-2 | Isoform 2 of Protein kinase C gamma type | -0.836501268 |
| Q15233 | Non-POU domain-containing octamer-binding protein | -0.836501268 |
| Q16653-12 | Isoform 12 of Myelin-oligodendrocyte glycoprotein | -0.836501268 |
| Q16849-1 | Receptor-type tyrosine-protein phosphatase-like N | -0.836501268 |
| Q6KB66 | Keratin, type II cytoskeletal 80 | -0.836501268 |
| Q96HU8 | GTP-binding protein Di-Ras2 | -0.836501268 |
| Q9H4A3 | Serine/threonine-protein kinase WNK1 | -0.836501268 |
| Q9Y570 | Protein phosphatase methylesterase 1 | -0.836501268 |
| P02533 | Keratin, type I cytoskeletal 14 | -0.836501268 |
| P08779 | Keratin, type I cytoskeletal 16 | -0.836501268 |
| Q7Z3Y9 | Keratin, type I cytoskeletal 26 | -0.836501268 |
| P08247 | Synaptophysin | -0.862496476 |
| P11234 | Ras-related protein Ral-B | -0.862496476 |
| P41732 | Tetraspanin-7 | -0.862496476 |
| Q15904 | V-type proton ATPase subunit S1 | -0.862496476 |
| Q92561 | phytanoyl-CoA hydroxylase-interacting protein | -0.862496476 |
| P08247-2 | Isoform 2 of Synaptophysin | -0.888968688 |
| P17677-1 | Neuromodulin | -0.888968688 |
| P56378-1 | 6.8 kDa mitochondrial proteolipid | -0.888968688 |
| Q969L2 | protein MAL2 | -0.888968688 |
| Q9UJZ1 | Stomatin-like protein 2, mitochondrial | -0.888968688 |
| P11169 | Solute carrier family 2, facilitated glucose transporter member 3 | -0.915935735 |
| P27449 | V-type proton ATPase 16 kDa proteolipid subunit | -0.915935735 |
| P51553-1 | isocitrate dehydrogenase [NAD] subunit gamma, mitochondrial | -0.915935735 |
| P63215 | Guanine nucleotide-binding protein G(I)/G(S)/G(O) subunit gamma-3 | -0.915935735 |
| Q8TDB8-1 | Solute carrier family 2, facilitated glucose transporter member 14 | -0.915935735 |
| Q99719-1 | Septin-5 | -0.915935735 |
| P61981 | 14-3-3 protein gamma | -0.971430848 |
| Q15818 | Neuronal pentraxin-1 | -0.971430848 |
| Q8NHG7 | Small VCP/p97-interacting protein | -0.971430848 |
| P13591-6 | Isoform 6 of Neural cell adhesion molecule 1 | -1 |
| Q6P3X3 | tetratricopeptide repeat protein 27 | -1 |
| O43759-3 | Isoform 1C of Synaptogyrin-1 | -1.029146346 |
| O95670-2 | Isoform 2 of V-type proton ATPase subunit G 2 | -1.029146346 |
| Q9BS92 | Protein NipSnap homolog 3B | -1.029146346 |
| P0CG38 | POTE ankyrin domain family member I | -1.120294234 |
| P0CG39 | POTE ankyrin domain family member J | -1.120294234 |
| Q6S8J3 | POTE ankyrin domain family member E | -1.120294234 |
| Q99447-1 | ethanolamine-phosphate cytidylyltransferase | -1.120294234 |
| Q9BYX7 | Putative beta-actin-like protein 3 | -1.120294234 |
| Q13813-3 | Isoform 3 of Spectrin alpha chain, non-erythrocytic 1 | -1.152003093 |
| P01859 | Ig gamma-2 chain C region | -1.251538767 |
| Q15833-1 | Syntaxin-binding protein 2 | -1.251538767 |
| P02686-1 | myelin basic protein | -1.434402824 |
| Q05682-1 | Caldesmon | -1.473931188 |
| O60493-1 | sorting nexin-3 | -1.514573173 |
| O14983-3 | Isoform 3 of Sarcoplasmic/endoplasmic reticulum calcium ATPase 1 | -1.556393349 |
| O14983 | Sarcoplasmic/endoplasmic reticulum calcium ATPase 1 | -1.785875195 |
| P20848 | Putative alpha-1-antitrypsin-related protein | -2.120294234 |

**Supplementary Table 9. Ten most up- or downregulated proteins commonly and coherently regulated across all genomic FTD etiologies**. For each significantly regulated protein the official Gene Symbol, Protein Description and the Log_2_ transforms of the iTRAQ expression ratios are represented. Proteins displayed in red or green show protein expression levels that were all up- or downregulated compared to control individuals, respectively. Proteins were ranked by the highest or lowest mean across the three genomic etiologies. The complete table of up- or downregulated proteins is available upon request.

**Supplementary Table 10. Ten most up- or downregulated proteins significantly and uniquely regulated in *FLNC* p.V831I patient compared to control individuals**. For each significantly regulated protein the official Gene Symbol, Protein Description and the Log_2_ transforms of the iTRAQ expression ratios are represented. Proteins displayed in red or green show protein expression levels that were all up- or downregulated compared to control individuals, respectively. The complete table of up- or downregulated proteins is available upon request.

**Supplementary Table 11. Ten most up- or downregulated proteins significantly and uniquely regulated in GRN p.0(IVS1+5G>C patients compared to control individuals**. For each significantly regulated protein the official Gene Symbol, Protein Description and the Log_2_ transforms of the iTRAQ expression ratios are represented. Proteins displayed in red or green show protein expression levels that were all up- or downregulated compared to control individuals, respectively. The complete table of up- or downregulated proteins is available upon request.

**Supplementary Table 12. Ten most up- or downregulated proteins significantly and uniquely regulated in *VCP* p.R159H patients compared to control individuals**. For each significantly regulated protein the official Gene Symbol, Protein Description and the Log_2_ transforms of the iTRAQ expression ratios are represented. Proteins displayed in red or green show protein expression levels that were all up- or downregulated compared to control individuals, respectively. The complete table of up- or downregulated proteins is available upon request.

**Supplementary Table 13a-d . Top 10 of word frequency scores for FTD-common and FlnC-, Grn- and Vcp-only higher-order word clouds.** Word frequencies from the cloud input data were calculated using WriteWords (<http://www.writewords.org.uk/word_count.asp>). Complete individual lists of word frequency scores for (**a**) FTD-common or (**b**) FLNC-, (**c**) GRN- and (**d**) VCP-only higher-order word clouds are available upon requested.

**a**

**b**

**c**

**d**

**Tables available upon request**

**Supplemental Table 9.** Complete table of up- or downregulated proteins commonly and coherently regulated across all genomic FTD etiologies.

**Supplemental Table 10.** Complete table of up- or downregulated proteins significantly and uniquely regulated in *FLNC* p.V831I patient compared to control individuals.

**Supplemental Table 11.** Complete table of up- or downregulated proteins significantly and uniquely regulated in *GRN* p.0(IVS1+5G>C) patients compared to control individuals.

**Supplemental Table 12.** Complete table of up- or downregulated proteins significantly and uniquely regulated in *VCP* p.R159H patients compared to control individuals.

**Supplemental Table 13a.** Complete table of word frequency scores for FTD-common higher-order word clouds.

**Supplemental Table 13b.** Complete table of word frequency scores for FLNC-only higher-order word clouds.

**Supplemental Table 13c.** Complete table of word frequency scores for GRN-only higher-order word clouds.

**Supplemental Table 13d.** Complete table of word frequency scores for VCP-only higher-order word clouds.

**Supplemental Table 14.** User-defined latent semantic indexing-based analysis of FLNC-specific proteins.

**Supplemental Table 15.** User-defined latent semantic indexing-based analysis of GRN-specific proteins.

**Supplemental Table 16.** User-defined latent semantic indexing-based analysis of VCP-specific proteins.

**Supplemental Table 17.** NIH DAVID GO term and KEGG Pathway analysis of the FTD-common protein dataset.

**Supplemental Table 18.** NIH DAVID GO term and KEGG Pathway analysis of the FLNC-only protein dataset.

**Supplemental Table 19.** NIH DAVID GO term and KEGG Pathway analysis of the GRN-only protein dataset.

**Supplemental Table 20.** NIH DAVID GO term and KEGG Pathway analysis of the VCP-only protein dataset.
