# Supplementary material for: Investigating the role of filamin C in Belgian patients with frontotemporal dementia linked to GRN deficiency in FTLD-TDP brains
Source: Acta Neuropathol Commun. 2015 Nov 10;3:68. doi: 10.1186/s40478-015-0246-7 (PMC4641381; doi:10.1186/s40478-015-0246-7)
Supplement: Additional file 2: Supplementary figures. — (DOC 2024 kb) [file 40478_2015_246_MOESM2_ESM.doc]

### **Supplementary Figures**


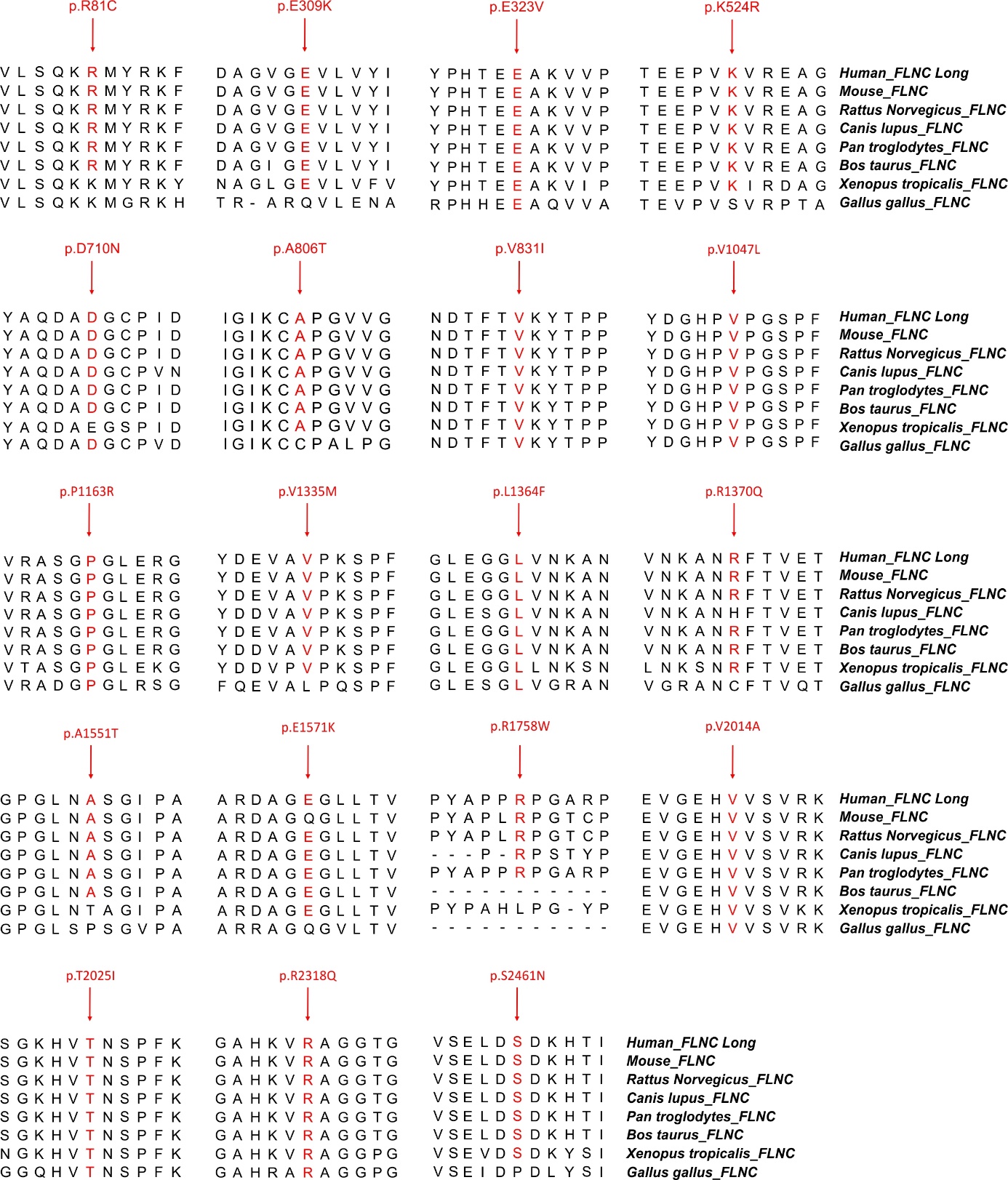


**Supplementary Fig. 1. Visualization of protein sequence alignment of identified patient-specific *FLNC* variants across different species.** Protein sequence alignment for patient-specific variants identified in *FLNC* shows evolutionary conservation across different species. The alignment is based on the long isoform of FLNC (NP_001449.3).


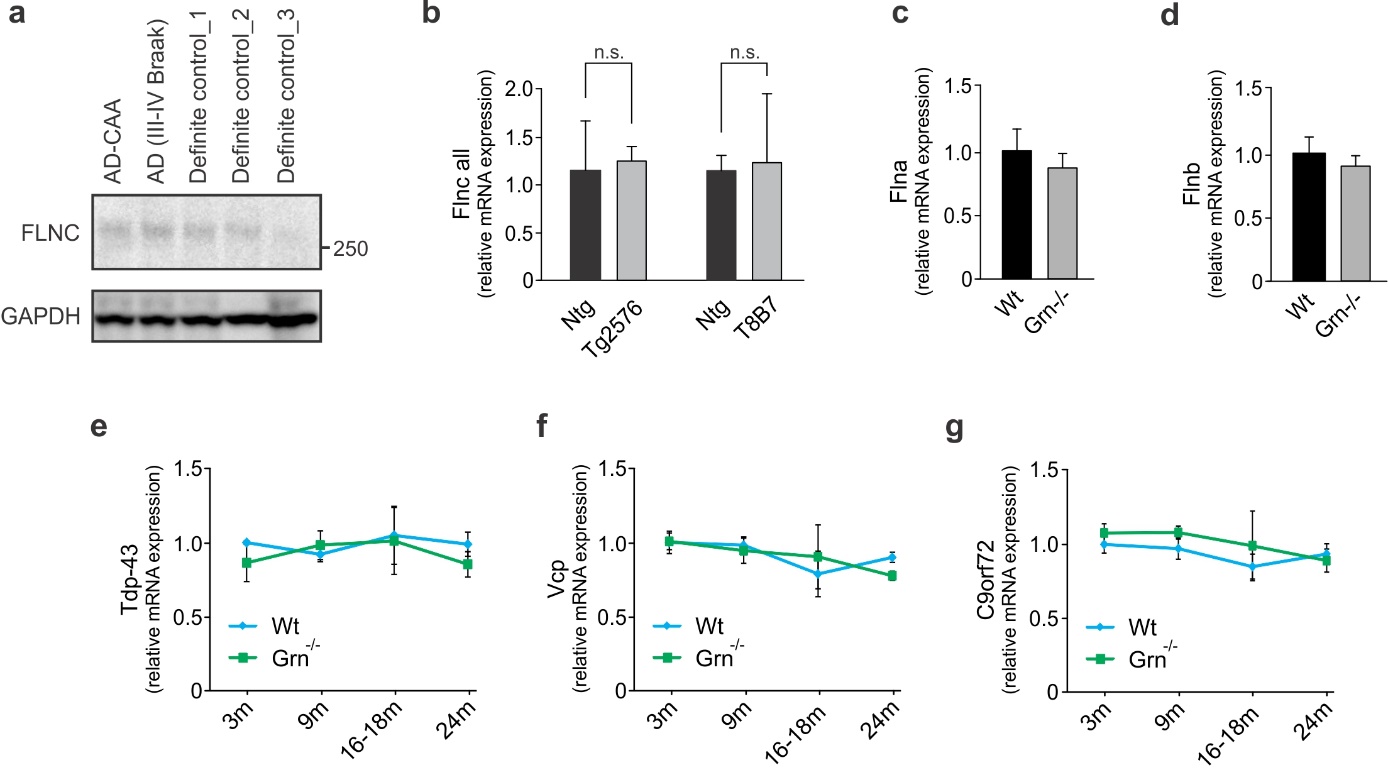


**Supplementary Fig. 2 Expression studies in mouse models and Alzheimer disease patients.** (**a**) Western blot analysis of frontal cortex of AD patients did not show strong alterations in FLNC expression compared to control individuals (**b**) No differences in *FLNC* transcript levels could be observed in two mouse models for Alzheimer’s disease (AD) overexpressing either the Swedish *APP* mutation (Tg2576) or the *PSEN1* p.G384A mutation (T8B7). (**c-d**) qRT-PCR analysis of mRNA expression levels of (**c**) mouse filamin A (*FlnA*) and (**d**) mouse filamin B (*FlnB*) measured in 24-month-old Grn^-/-^ mice compared to wild-type (Wt) littermates. (**e-g**) qRT-PCR analysis of mRNA expression levels of FTD-associated genes including (**e**) *Tardbp*, (**f**) *Vcp* and (**g**) *C9orf72* in Grn^-/-^ mice of different ages compared to Wt littermates. *n.s. not significant*


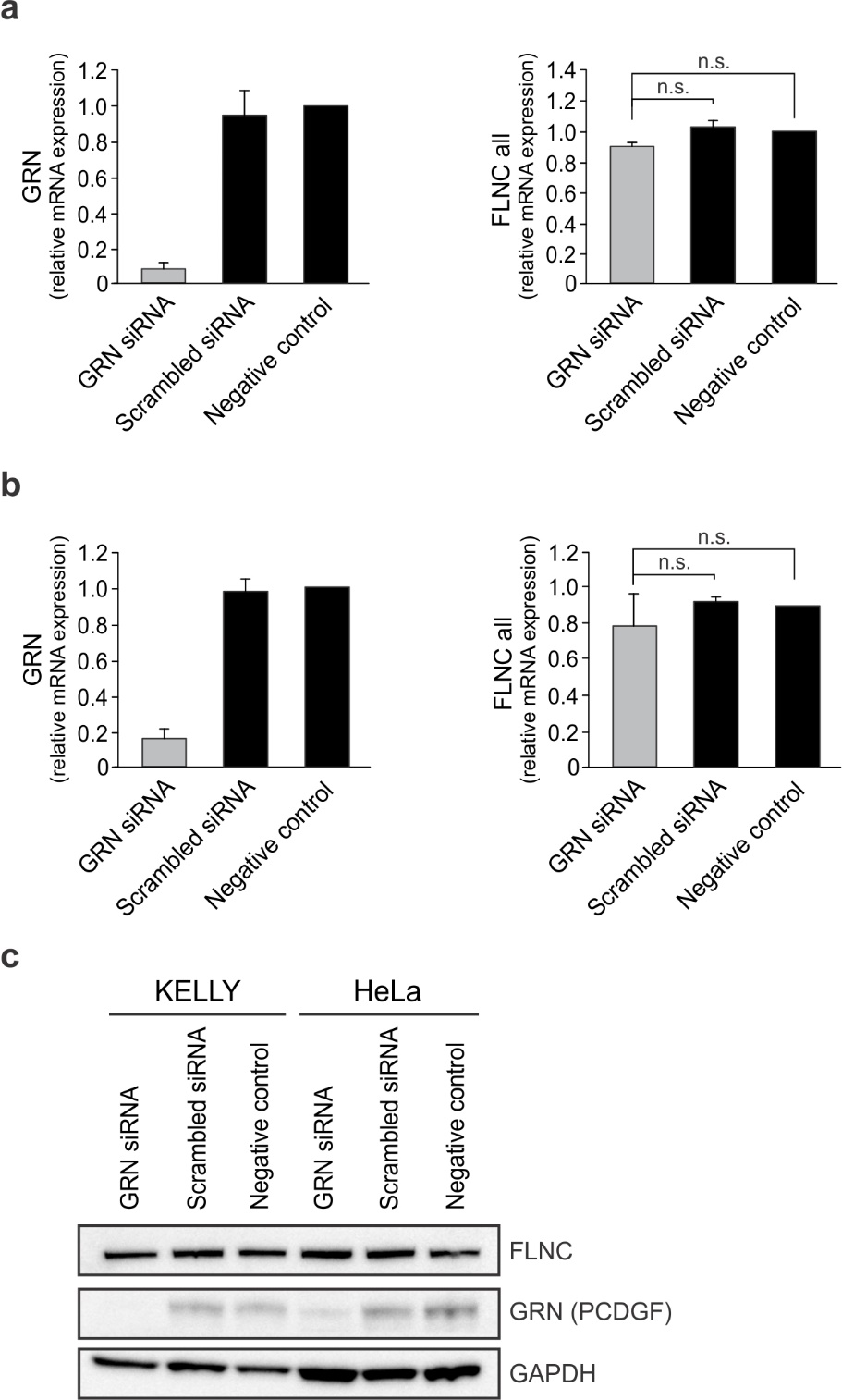


**Supplementary Fig. 3** (**a-b**) qRT-PCR and (**c**) Western blot analysis on (**a**) human cervical carcinoma cells (HeLa) and (**b**) human neuroblastoma (KELLY) cells showed strong reductions of progranulin (*GRN*) (**a-b**, left panels), but no alterations in *FLNC* transcript levels (**a-b**, right panels). (**c**) Western blot analysis confirmed the reduced GRN levels and the unaltered FLNC levels on protein level. *n.s. not significant*


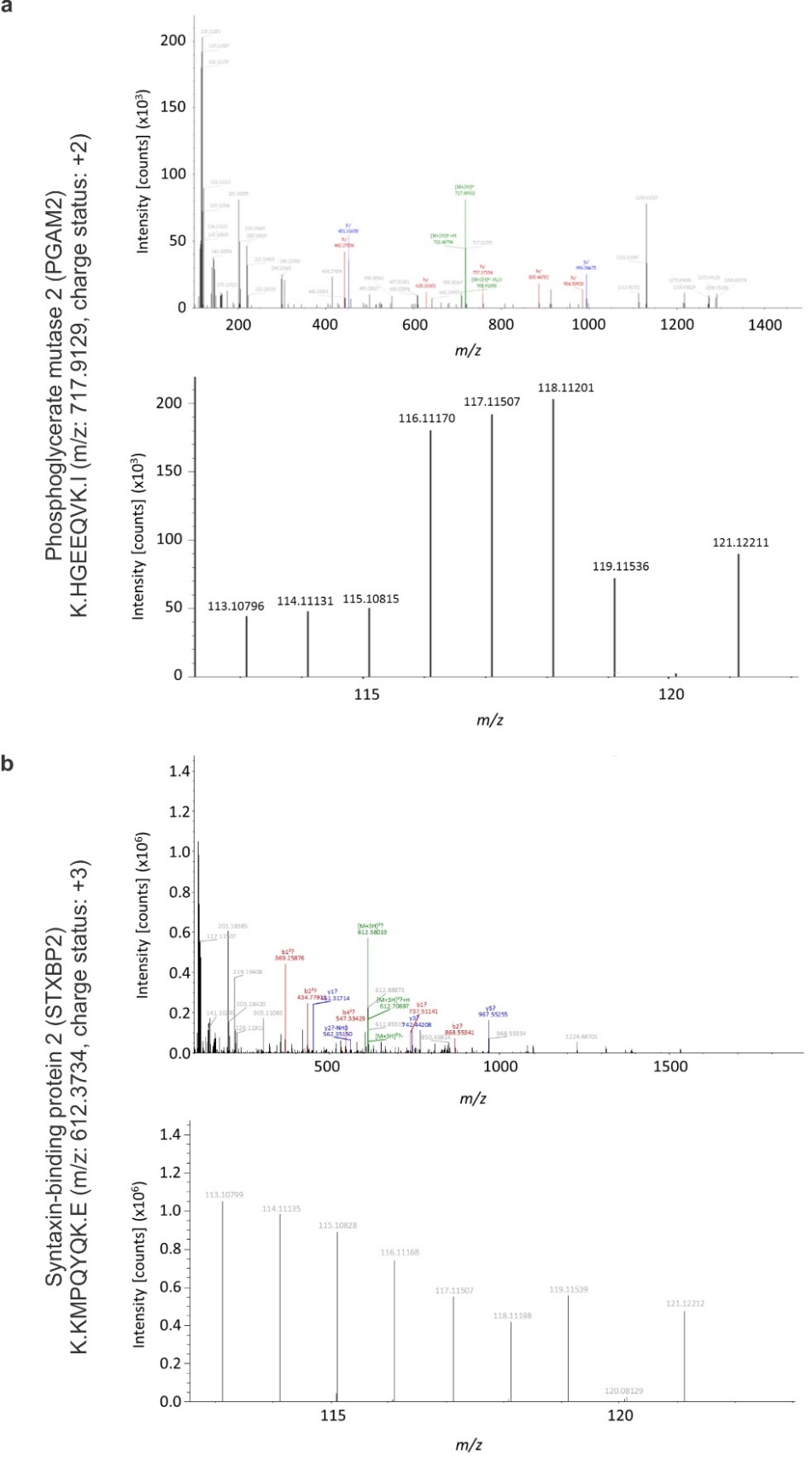


**Supplementary Fig. 4. Mass spectrometry chromatograms of validated target proteins.** Example of a chromatogram of (**a**, upper panel) PGAM2 and (**b**, upper panel) STXBP2 obtained by MS. Chromatograms showing the reporter ions detected in the spectrum for (**a**, lower panel) PGAM2 and (**b**, lower panel) STXBP2 demonstrating the different abundancies for the iTRAQ labels used.


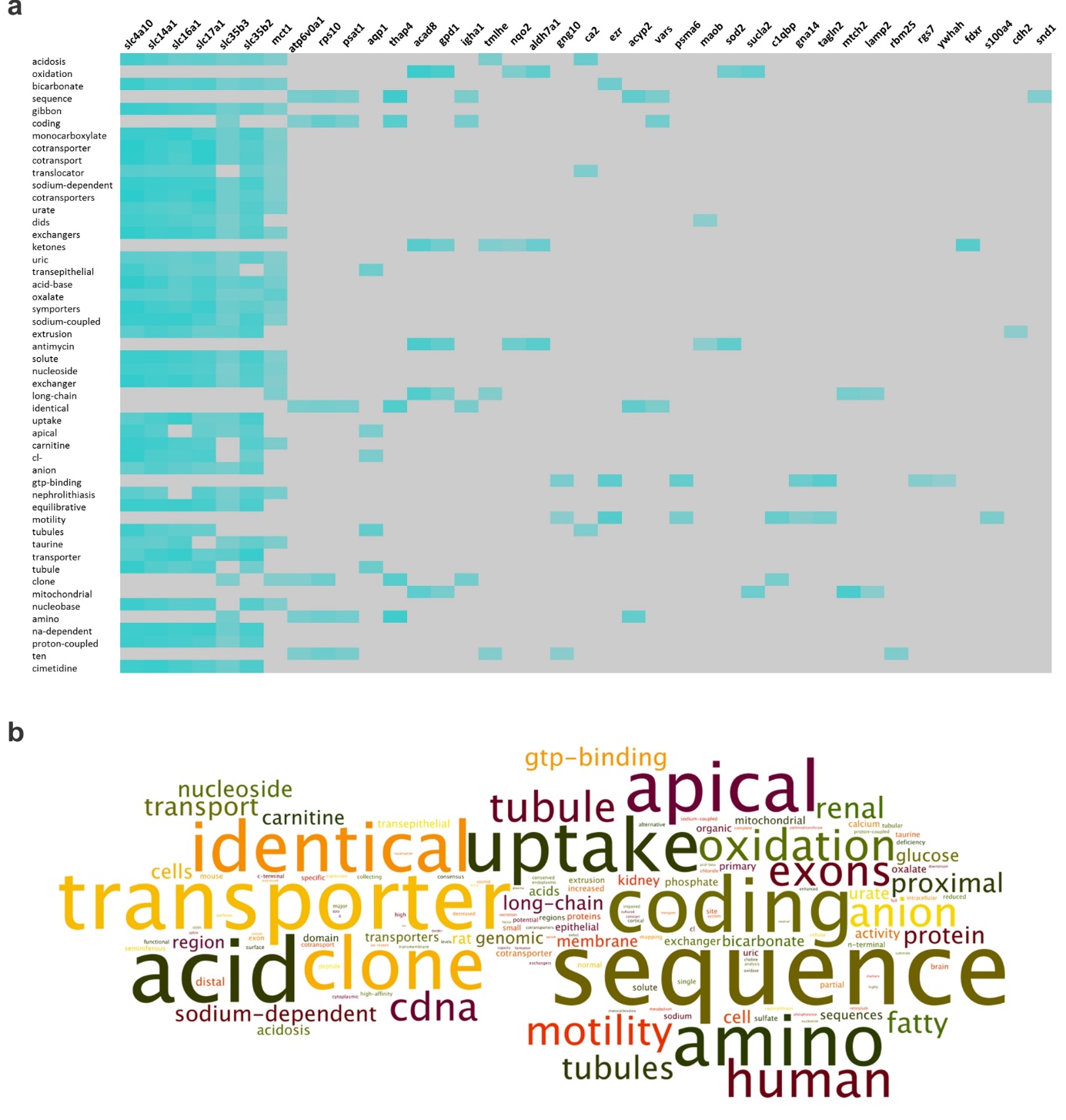


**Supplementary Fig. 5. Representation of the *GRN* p.0(IVS1+5G>C) unique protein dataset using *Textrous!* and word clouds** (**a**) Analysis of the *GRN* p.0(IVS1+5G>C) unique protein dataset using *Textrous!* natural language processing (NLP). Strongest correlations between words (vertical) and proteins (horizontal) are presented in a *Textrous!* heat map. Teal-colored blocks indicate strongly-associated gene-word interactions in an intensity-sensitive manner; grey blocks indicate no significant interaction (**b**) Word clouds obtained from Wordle (<http://www.wordle.net/>) analyzing the *Textrous!* output unique for the *GRN* p.0(IVS1+5G>C) mutation.


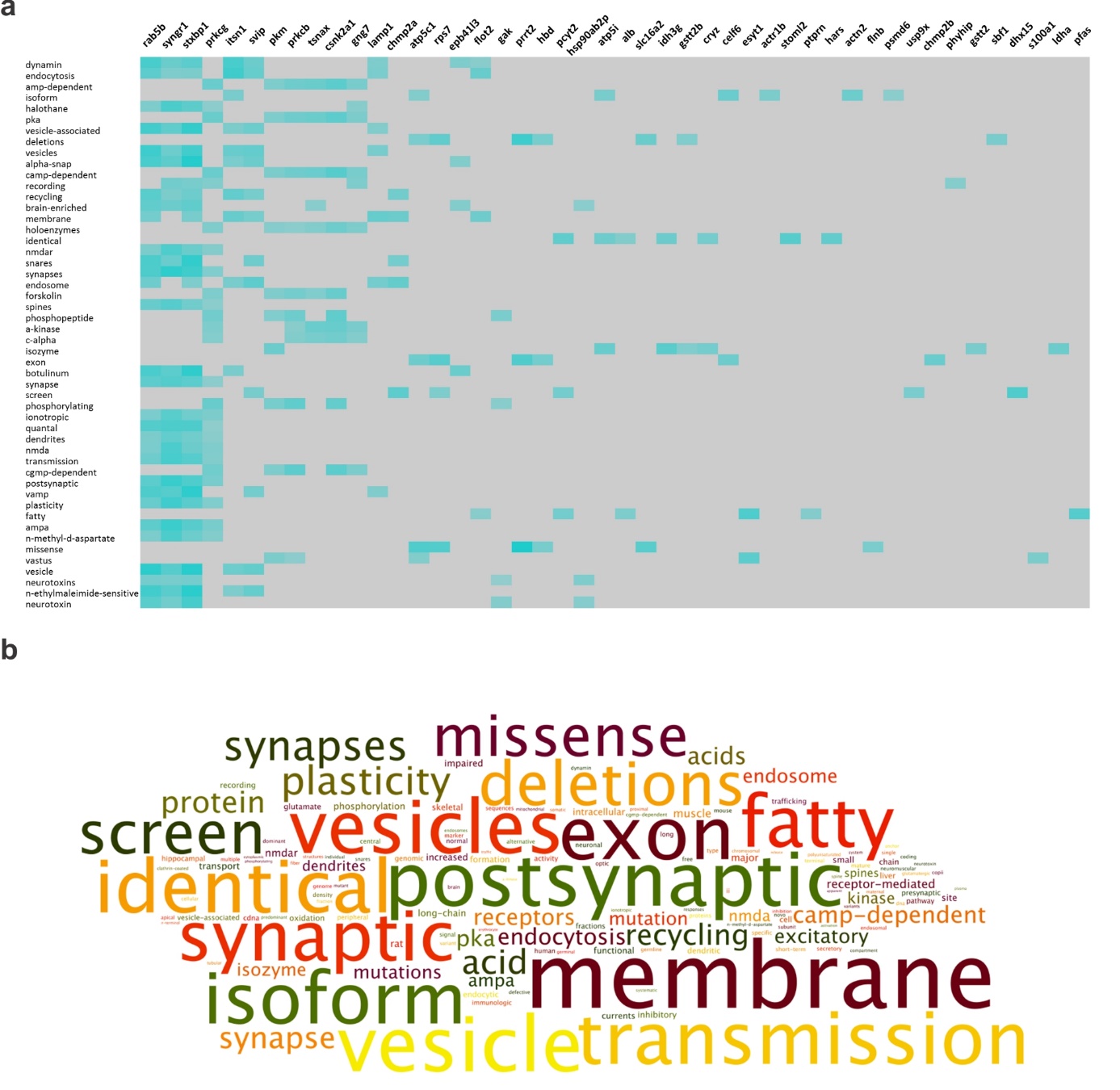


**Supplementary Fig. 6** **Representation of the *VCP* p.R159H unique protein dataset using Textrous! and word clouds** (**a**) Analysis of the *VCP* p.R159H unique protein dataset using *Textrous!* natural language processing (NLP). Strongest correlations between words (vertical) and proteins (horizontal) are presented in a *Textrous!* heat map. Teal-colored blocks indicate strongly-associated gene-word interactions in an intensity-sensitive manner; grey blocks indicate no significant interaction (**b**) Word clouds obtained from Wordle (<http://www.wordle.net/>) analyzing the *Textrous!* output unique for the *VCP* p.R159H mutation.
